# Tumour cell-derived Wnt7a recruits and activates fibroblasts to promote tumour aggressiveness

**DOI:** 10.1038/ncomms10305

**Published:** 2016-01-18

**Authors:** Alexandra Avgustinova, Marjan Iravani, David Robertson, Antony Fearns, Qiong Gao, Pamela Klingbeil, Andrew M. Hanby, Valerie Speirs, Erik Sahai, Fernando Calvo, Clare M. Isacke

**Affiliations:** 1 The Breast Cancer Now Toby Robins Research Centre, The Institute of Cancer Research, 237 Fulham Road, London SW3 6JB, UK; 2 Leeds Institute of Cancer and Pathology, University of Leeds, Leeds LS9 7TF, UK; 3 Tumour Cell Biology Laboratory, Francis Crick Institute, 44 Lincoln's Inn Fields, London WC2A 3LY, UK; 4 Tumour Microenvironment Team, The Institute of Cancer Research, 237 Fulham Road, London SW3 6JB, UK

## Abstract

Stromal fibroblast recruitment to tumours and activation to a cancer-associated fibroblast (CAF) phenotype has been implicated in promoting primary tumour growth and progression to metastatic disease. However, the mechanisms underlying the tumour:fibroblast crosstalk that drive the intertumoural stromal heterogeneity remain poorly understood. Using *in vivo* models we identify Wnt7a as a key factor secreted exclusively by aggressive breast tumour cells, which induces CAF conversion. Functionally, this results in extracellular matrix remodelling to create a permissive environment for tumour cell invasion and promotion of distant metastasis. Mechanistically, Wnt7a-mediated fibroblast activation is not dependent on classical Wnt signalling. Instead, we demonstrate that Wnt7a potentiates TGFβ receptor signalling both in 3D *in vitro* and *in vivo* models, thus highlighting the interaction between two of the key signalling pathways in development and disease. Importantly, in clinical breast cancer cohorts, tumour cell Wnt7a expression correlates with a desmoplastic, poor-prognosis stroma and poor patient outcome.

## Abstract (web summary)

How cancer-associated fibroblasts become activated to support metastasis is not well understood. Here, Avgustinova *et al*. show that tumour cell-derived Wnt7a activates TGFβ signaling in fibroblasts, inducing them to remodel the microenvironment to the state, which promotes tumour invasiveness and metastasis.

## Graphical abstract

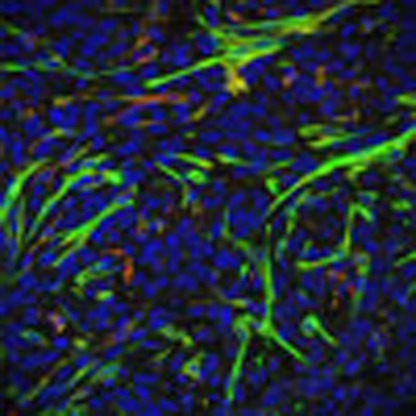

Fibroblasts constitute a significant proportion of the stromal compartment in many solid tumours and these infiltrating cells can acquire an activated cancer-associated fibroblast (CAF) phenotype. There is now extensive evidence functionally implicating CAFs in tumour progression via their ability to deposit and remodel extracellular matrix components, secrete pro-tumorigenic factors and modulate the immune compartment^1^,^2^,^3^,^4^,^5^. In breast cancer this so-called ‘desmoplastic response' shows a clinical correlation with invasion and poor patient prognosis^6^. In addition, there is an increasing body of data supporting a role of CAFs in promoting resistance to chemotherapy and targeted agents^7^. Despite the growing interest in the functional role of CAFs in tumours, much of their biology remains a mystery because of the lack of specific markers, as well as fibroblast phenotypic plasticity and heterogeneity both *in vivo* and *in vitro*^8^,^9^. Together, these have hampered efforts to disentangle the mechanisms underlying tumour:fibroblast crosstalk that could reveal novel strategies for disrupting stromal activation and thereby enhancing therapeutic targeting of tumour cells. In particular, although it is well recognized that CAF recruitment and activation are strikingly variable between different breast cancers^10^,^11^,^12^, there is little understanding of the mechanisms driving this intertumour stromal heterogeneity.

The goal of this study was to identify the mechanisms that underpin the differential infiltration and activation of CAFs in breast cancer and to determine the relevance of these observations to human disease. By gene expression profiling of aggressive and non-aggressive tumours, we identify Wnt7a as a key tumour cell-secreted factor that promotes fibroblast recruitment and activation in three-dimensional (3D) *in vitro* assays and *in vivo*. Mechanistically, Wnt7a does not signal through the classical Wnt pathways to trigger CAF conversion but potentiates TGFβ receptor signalling. Interestingly, this promotion of TGFβ receptor signalling is dependent on dynamic reciprocity between fibroblasts and a malleable extracellular matrix. Functionally, high Wnt7a expression in tumour cells promotes metastasis *in vivo* and, in human breast cancers, correlates with a desmoplastic, poor-prognosis stroma with high fibroblast TGFβ pathway activation and reduced patient survival. We identify a novel level of interaction between Wnt and TGFβ pathways in CAFs, which presents a potential avenue for inhibiting or reversing the production of a tumour-promoting stroma.

## Results

### Stromal heterogeneity in a breast cancer progression model

**Figure 1:**
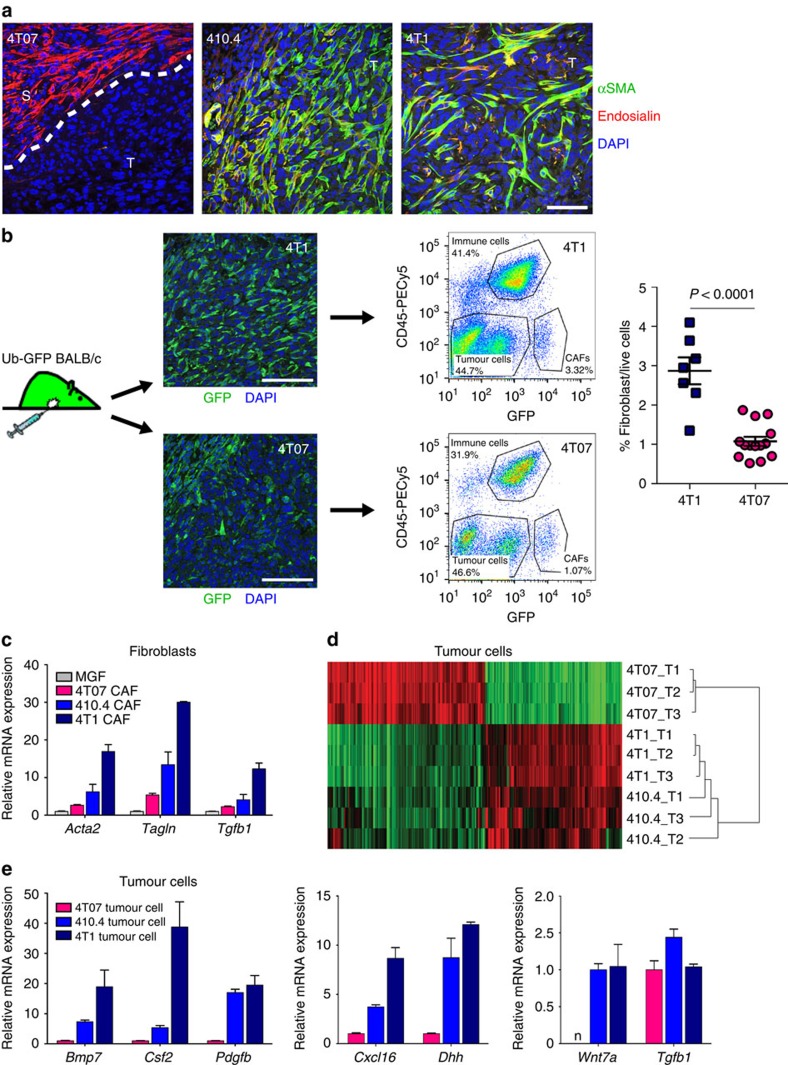
Differential CAF recruitment and activation *in vivo*. (**a**) 4T07, 410.4 and 4T1 orthotopic tumours in Balb/c mice were formalin-fixed and paraffin-embedded and sections stained with FITC–αSMA (green) and endosialin followed by Alexa 555-anti-rabbit-IgG (red). Nuclei were counterstained with 4,6-diamidino-2-phenylindole (DAPI; blue). Representative confocal images are shown. Dashed line indicates the border between tumour, T, and stroma, S. Scale bar, 75 μm. Low-power images are shown in Supplementary Fig. 1a. (**b**) Isolation of tumour cells and CAFs. 4T07, 410.4 and 4T1 cells were inoculated into the fourth mammary fat pad of Ub-GFP Balb/c mice. Confocal images of 4T1 and 4T07 tumours showing GFP-negative tumour cells and GFP-positive stromal cells. Scale bar, 25 μm. Tumours were dissociated and subject to FACSorting. Shown are representative FACS profiles from 4T1 and 4T07 tumours. Right panel shows the number of GFP+; CD45− fibroblasts as a percentage of live cells in individual 4T07 (*n*=14) and 4T1 (*n*=7) tumours. Comparison made using Student's *t*-test. (**c**,**d**) Tumour cells and fibroblasts from three independent isolates were directly lysed for RNA extraction. (**c**) *Acta2*, *Tagln* and *Tgfb1* mRNA expression in normal MGFs and CAFs monitored using qPCR. Data shown are the mean±s.e.m. relative quantification (RQ) values from three independent biological replicates. (**d**) Tumour cells were subject to whole-genome expression profiling. Dendrogram shows correlation-centred hierarchical clustering based on average linkage. Shown are tumour cell expression data of probes significantly differentially expressed between 410.4/4T1 and 4T07 tumour cells with a fold change >2 (498 probes). (**e**) qPCR validation of selected genes from independently FACSorted tumour cell samples. n, non-detectable. Data shown are the mean±s.e.m. RQ values from three independent biological replicates.

In this study we employed the 4T1 series of mouse mammary carcinoma tumours as an *in vivo* model of breast cancer progression. The 4T1 series cell lines have a single origin but, despite all giving rise to primary tumours in syngeneic Balb/c mice, differ in their metastatic potential^13^,^14^,^15^. To characterize their *in vivo* stromal phenotypes, orthotopic tumours were first stained with the pan-fibroblast marker endosialin^16^ and the fibroblast activation marker αSMA. Strikingly, we found that infiltrating αSMA-positive CAFs are abundant in the metastatic 4T1 and 410.4, but not in the less aggressive 4T07 tumours (Fig. 1a and Supplementary Fig. 1a). As both endosialin and αSMA are also expressed by tumour pericytes^17^, sections were also stained with the endothelial marker endomucin. The low incidence of endosialin-positive cells associated with endomucin-stained blood vessels indicates that the infiltrating endosialin-positive cells are predominantly of fibroblast identity (Supplementary Fig. 1b). As the goal of this project was to interrogate tumour:stroma crosstalk *in vivo*, 4T1, 410.4 and 4T07 cells were inoculated orthotopically into Ub-GFP Balb/c mice. This approach allowed tumour cell and CAF populations to be isolated by FACSorting and directly lysed for gene expression profiling (Fig. 1b). In concordance with the histological data (Fig. 1a), we show that aggressive 4T1 tumours contain a significantly higher proportion of CAFs (GFP+; CD45− cells) as compared with the less aggressive 4T07 tumours (Fig. 1b). qPCR analysis of fibroblast populations confirmed that CAFs from more aggressive breast tumours have an ‘activated' CAF phenotype as evidenced by the gradient increase in expression of the myofibroblast markers αSMA (*Acta2*)^2^, transgelin (*Tagln*)^18^ and TGFβ1 (*Tgfb1*)^19^,^20^,^21^ from control mouse mammary gland fibroblasts (MGFs) to 4T07 fibroblasts to 410.4/4T1 fibroblasts (Fig. 1c). To explore the cause of this differential regulation, the gene expression profiles of the tumour cells isolated from the less aggressive 4T07 tumours with low-level CAF activation and from the more aggressive 410.4 and 4T1 tumours characterized by substantial CAF recruitment and activation were interrogated. When subjected to hierarchical clustering, the 4T07 tumour cells clearly separate away from 410.4 and 4T1 tumour cells (Fig. 1d). A pairwise comparison between the two groups identified 3,406 differentially expressed genes, with 498 probes showing a fold change >2. We hypothesized that CAF recruitment and activation is, at least initially, driven by paracrine signalling, and therefore the main focus of our further investigations was differentially expressed soluble factors. Functional annotation of the differentially expressed genes identified 56 secreted factors that are more highly expressed by 410.4 and 4T1 tumour cells compared with 4T07 tumour cells (Supplementary Table 1) and of potential interest for further analysis.

### Tumour cell-secreted Wnt7a promotes fibroblast activation

After bioinformatic analysis and extensive literature review, we selected a range of tumour cell-secreted factors for further investigation. qPCR validation using additional independently FACSorted populations confirmed that all selected factors show lower expression in 4T07 compared with 410.4/4T1 tumour cell samples (Fig. 1e). Of note, we did not observe a differential tumour cell expression of TGFβ1, the secreted factor most commonly associated with myofibroblast conversion^1^,^2^ (Fig. 1e).

**Figure 2:**
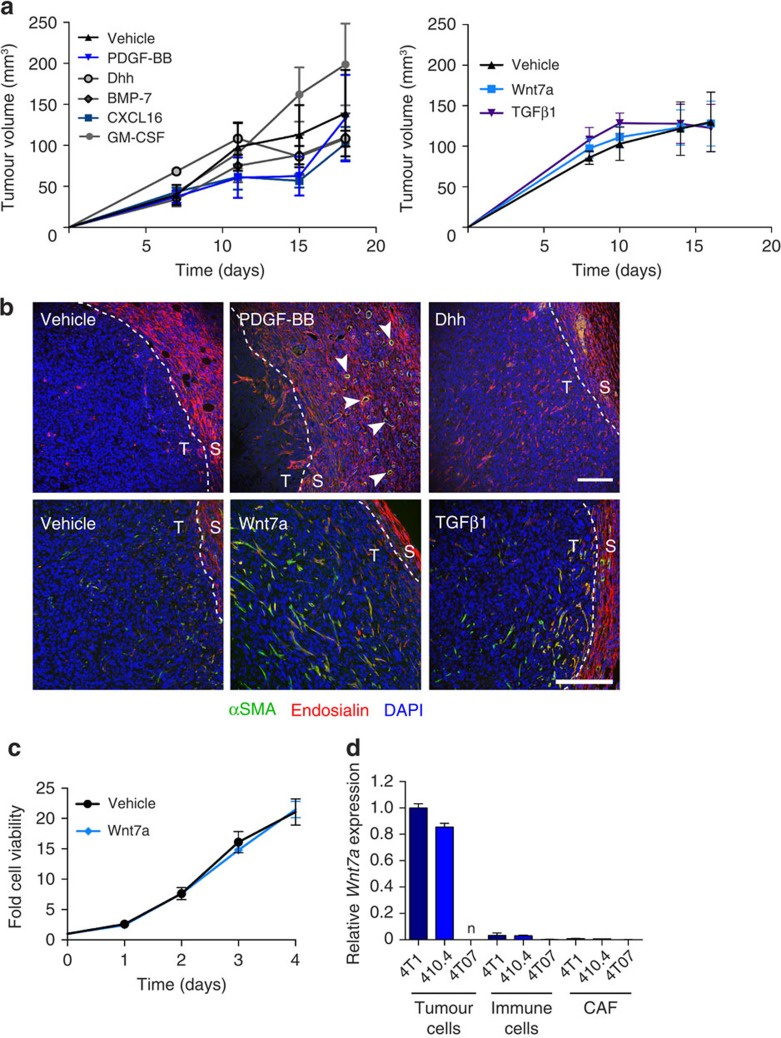
Wnt7a promotes fibroblast recruitment and activation *in vivo* and *in vitro.* (**a**,**b**) 4T07 cells were injected bilaterally into the fourth mammary fat pads of wild-type Balb/c mice (four to five mice per group) and treated with recombinant growth factors (see Methods section). (**a**) Data represent the mean tumour volume±s.e.m. (see Supplementary Fig. 2a for tumour weights). Groups were compared with two-way ANOVA followed by Bonferroni post-test. No statistically significant differences were found. (**b**) Tumours were stained for αSMA-FITC (green) and endosialin followed by Alexa 555-anti-rabbit-IgG (red). Nuclei were counterstained with DAPI (blue). Representative confocal images are shown. T, tumour; S, stroma; arrowheads, peritumoural blood vessels. Scale bar, 150 μm. See Supplementary Fig. 2b for separated images. (**c**) NF#1 fibroblasts were treated with vehicle or 100 ng ml^−1^ Wnt7a and viable cell amount monitored by CellTiter-Glo assay at the indicated time points. Data represent the mean values from four wells per condition at each time point±s.e.m. (**d**) Tumour (GFP−; CD45−), immune cell (GFP–; CD45–) and CAF (GFP–; CD45−) populations from 4T1, 410.4 and 4T07 tumours. *Wnt7a* mRNA expression was monitored using qPCR as described in **b**. n, non-detectable. Data shown are the mean±s.e.m. RQ values from two independent biological replicates.

To assess the ability of these factors to promote fibroblast recruitment and activation *in vivo*, six targets were selected and 4T07 tumours were supplemented with the corresponding recombinant proteins by intratumoural injection. Supplementation had no effect on tumour take, tumour growth (Fig. 2a) or tumour weight at necroscopy (Supplementary Fig. 2a). As observed in untreated tumours (Fig. 1a), vehicle-treated 4T07 tumours have few, if any, infiltrating CAFs and the peritumoural fibroblasts remain αSMA-negative (Fig. 2b and Supplementary Fig. 2b). No differences are observed in the stromal compartments of BMP-7, CXCL16 or granulocyte–macrophage colony-stimulating factor (GM-CSF)-treated tumours (data not shown). Dhh and PDGF-BB treatment results in increased fibroblast infiltration; however, the infiltrating fibroblasts remain αSMA-negative suggesting that these factors have some ability to promote fibroblast recruitment but not activation. PDGF-BB treatment also stimulated a stromal angiogenic response (Fig. 2b). Strikingly, Wnt7a (Supplementary Fig. 2c) was as effective as TGFβ1 treatment in generating 4T07 tumours containing a substantial population of activated CAFs in the tumour core (Fig. 2b). The observation that Wnt7a has no effect on fibroblast proliferation *in vitro* (Fig. 2c) indicates that the increase in intratumoural fibroblasts results from increased fibroblast recruitment and is not solely due to mitotic expansion.

While our data show that aggressive tumour cells secrete Wnt7a, we next sought to determine whether there are other sources of Wnt7a within the tumour. qPCR analysis of different cell populations freshly isolated from the tumours demonstrates that indeed aggressive tumour cells are the principal sources of Wnt7a, with little or no expression detected in CAFs, immune cell populations or less aggressive 4T07 tumour cells (Fig. 2d).

**Figure 3:**
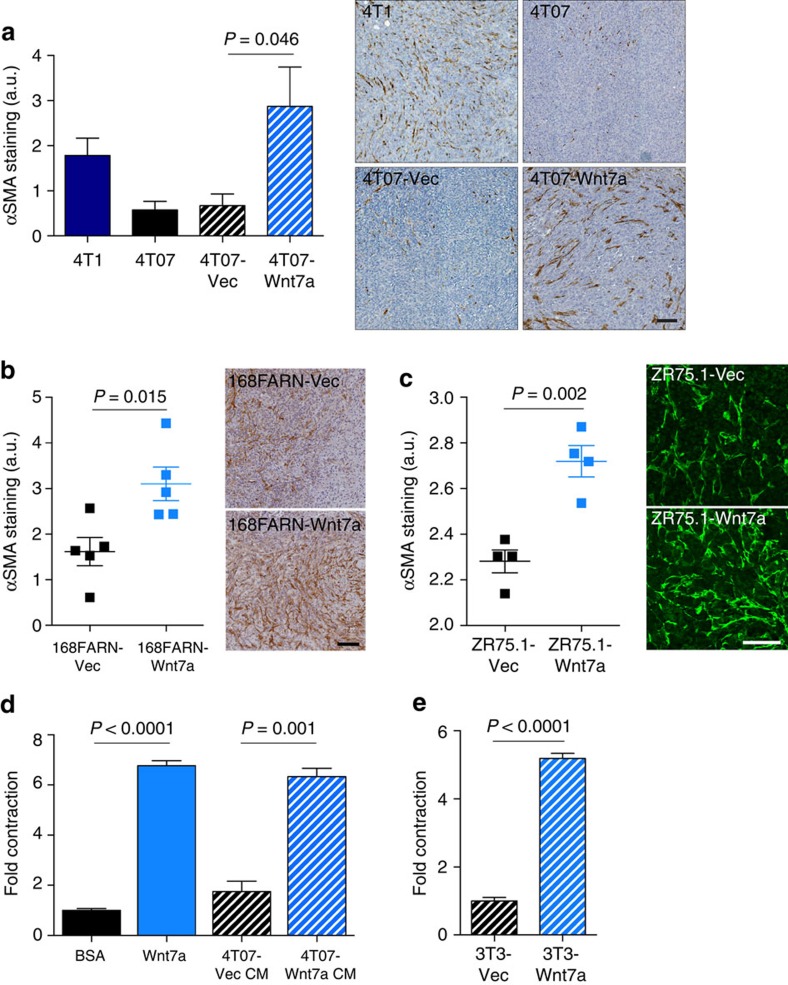
Wnt7a promotes fibroblast recruitment and activation *in vivo* and *in vitro.* Quantification of αSMA staining in primary tumours following ectopic expression of Wnt7a (see Supplementary Fig. 3 for qPCR analysis). Left panels show quantification of αSMA staining. Right panels show representative images of αSMA staining. (**a**) Balb/c mice inoculated with 5 × 10^5^ parental 4T1, parental 4T07, vector-transfected 4T07 (4T07-Vec) and 4T07-expressing Wnt7a (4T07-Wnt7a) cells. Tumours were removed on day 30. Tumour numbers; 4T1, *n*=5; 4T07, 4T07-Vec, 4T07-Wnt7a, *n*=9 or 10. Scale bar, 100 μm. (**b**) Balb/c mice inoculated with 3 × 10^6^ 168FARN-Vec or 168FARN-Wnt7a cells. Tumours were removed on day 12. Data shown are from five mice per group. Scale bar, 100 μm. (**c**) 2 × 10^6^ ZR75.1-Vec and ZR75.1-Wnt7a were inoculated into nonobese diabetic severe combined immunodeficient mice. Tumours were removed on day 28. Data shown are from four mice per group. Scale bar, 50 μm. (**d**) NF#1 MGFs embedded in collagen gels were treated with BSA or Wnt7a (100 ng ml^−1^) or conditioned medium from 4T07 cells transfected with vector alone (4T07-Vec CM) or Wnt7a (4T07-Wnt7a CM) for 72 h. Data show quantification of matrix remodelling as monitored by gel contraction±s.e.m. Equivalent results were obtained in three independent experiments (**e**) NIH-3T3 fibroblasts expressing empty vector (3T3-Vec) or Wnt7a (3T3-Wnt7a) were assayed as described in **d**. All comparisons were made using Student's *t*-test.

Next, to assess the effect of manipulating tumour cell Wnt7a expression levels on the fibroblast compartment *in vivo*, CAF recruitment was assessed in tumours generated by orthotopic inoculation of 4T07 cells ectopically expressing Wnt7a (4T07-Wnt7a; Fig. 3a and Supplementary Fig. 3a). Compared with 4T07 cells infected with vector alone (4T07-Vec), 4T07-Wnt7a tumours show a significantly increased infiltration of activated CAFs into the tumour core, equivalent to that observed in 4T1 tumours. Finally, we extended this analysis to a non-metastatic member of the 4T1 cell line series, 168FARN (refs 13, 14, 15), and a human breast cancer cell line, ZR75.1. In both cases, ectopic expression of Wnt7a promotes a significant increase in recruitment of αSMA-positive CAFs (Fig. 3b,c and Supplementary Fig. 3b,c).

Finally, we tested whether the *in vivo* functional CAF conversion triggered by Wnt7a could be recapitulated in an *in vitro* assay. Increased fibroblast contractility is a major hallmark of CAF conversion and can be readily monitored by contraction of fibroblast-containing 3D collagen gels^22^. Treatment with either recombinant Wnt7a or conditioned medium from 4T07 cells ectopically expressing Wnt7a, but not conditioned medium from 4T07 cells transfected with vector alone, significantly enhanced normal fibroblast contractility (Fig. 3d). Moreover, fibroblasts ectopically expressing Wnt7a were more contractile than control fibroblasts expressing empty vector alone (Fig. 3e).

Together, these data highlight Wnt7a as a novel and highly potent tumour cell-secreted factor that is sufficient to drive conversion of fibroblasts into CAFs within the tumour microenvironment. We next investigated the underlying paracrine signalling events to better understand the molecular mechanisms of action of Wnt7a.

### Wnt7a-driven CAF conversion is TGFβ-signalling-dependent

**Figure 4:**
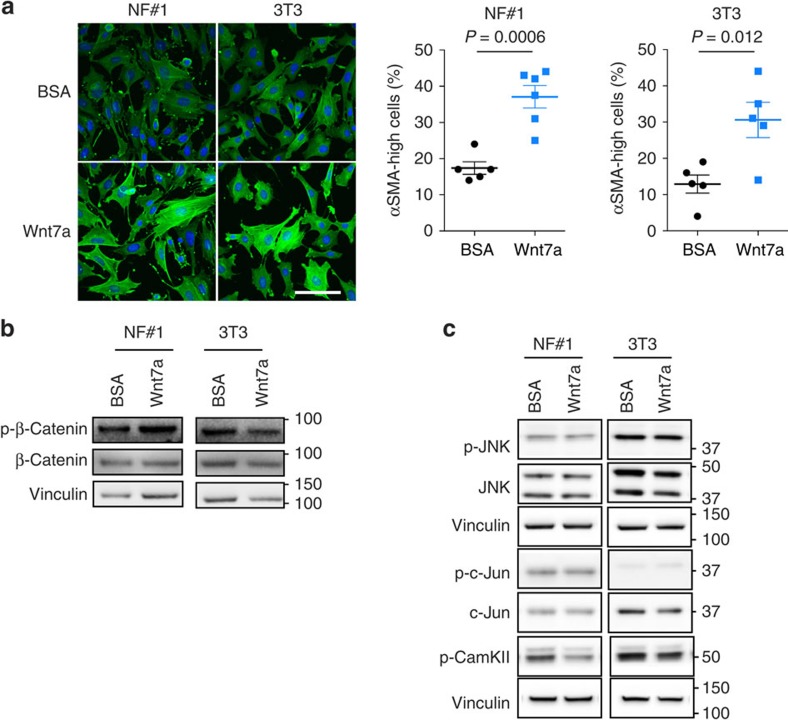
Wnt7a signalling in fibroblasts. NF#1 or NIH-3T3 fibroblasts were plated on Softwell hydrogels in serum-free Advanced DMEM. Twenty-four hours later, cells were treated with BSA or Wnt7a (100 ng ml^−1^) for 24 h. (**a**) Cells were fixed and stained for αSMA-FITC (green). Nuclei were counterstained with DAPI. Scale bar, 100 μm. Right panel, % cells with strong αSMA fibres±s.e.m. Comparison was made using Student's *t*-test. (**b**,**c**) Samples were subjected to immunoblotting with the indicated antibodies. Molecular size markers are in kDa.

Wnt7a has been demonstrated to play a role in development^23^,^24^,^25^, regeneration^26^, muscle satellite and neural^27^,^28^ stem cell expansion and malignant transformation^29^,^30^, underlining the importance of this Wnt family member in a multitude of biological processes. At the molecular level, Wnt7a has been implicated in both β-catenin-dependent^29^,^31^ and β-catenin-independent^32^,^33^ signalling, determined by cell and tissue context. To interrogate the Wnt7a signalling pathways operating in CAF activation, we used both immortalized NF#1 mouse MGFs and NIH-3T3 fibroblasts. Using cells plated on tissue culture plastic, we first assessed the activation of β-catenin-dependent Wnt signalling in response to Wnt7a. While, as expected, the GSK-3β inhibitor SB216763 or Wnt3a, a known inducer of β-catenin-dependent Wnt signalling, results in a robust translocation of β-catenin into the nucleus, Wnt7a did not activate this pathway in either NF#1 or NIH-3T3 (Supplementary Fig. 4a) fibroblasts. Similar results were obtained using the TOPflash Wnt reporter assay (Supplementary Fig. 4b). Next, to assess whether Wnt7a signals through either of the β-catenin-independent Wnt signalling pathways, the activation of key pathway components was examined using western immunoblot analysis. Strikingly, there was no evidence that Wnt7a activates either Wnt/Ca2+ or planar cell polarity signalling as monitored by CaMKII phosphorylation or c-Jun phosphorylation, respectively (Supplementary Fig. 4c). Importantly, in contrast to the ability of Wnt7a to drive fibroblast activation *in vivo* (Figs 2 and 3a–c) and in 3D collagen gels (Fig. 3d,e), we were unable to detect a Wnt7a-mediated increase in the αSMA expression in fibroblasts plated on rigid, nonphysiological tissue culture plastic (data not shown), suggesting that Wnt7a-mediated CAF conversion acts in concert with fibroblast mechanosensing. To address this, we established a more physiologically relevant model in which fibroblasts are plated on extracellular matrix-coated synthetic hydrogels. In this setting we are able to recapitulate a phenotypic Wnt7a-mediated CAF conversion of NF#1 and NIH-3T3 fibroblasts as assessed by increased αSMA levels and localization of αSMA into characteristic stress fibres (Fig. 4a). However, when plated on hydrogels, we again observe no activation of β-catenin, Ca2+-dependent or planar cell polarity Wnt signalling pathways (Fig. 4b,c).

**Figure 5:**
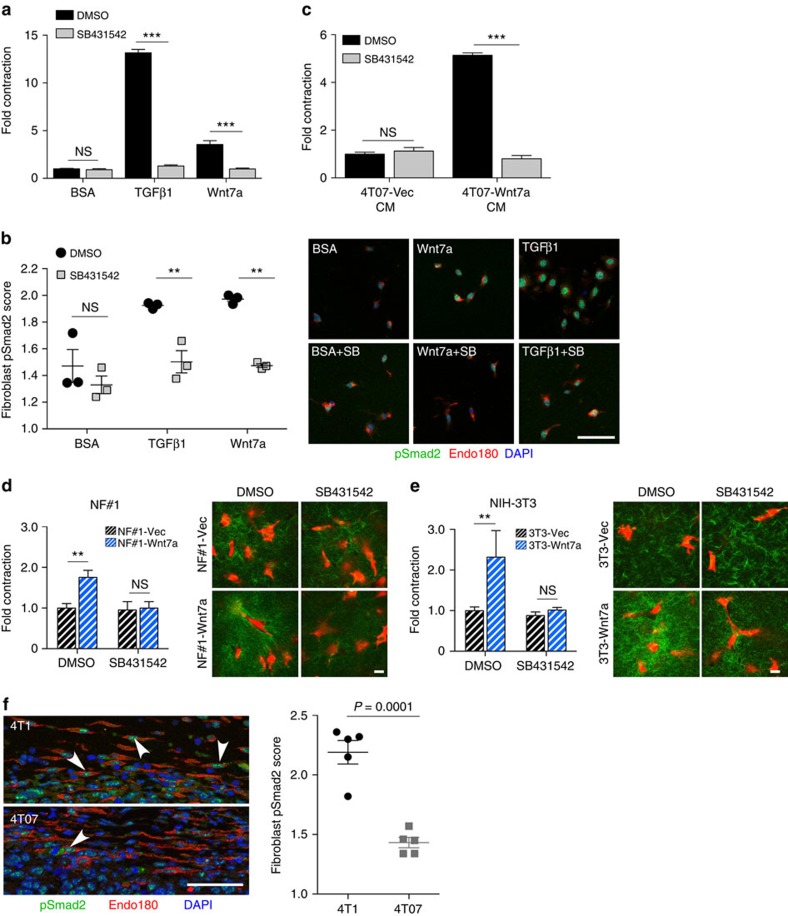
Wnt7a and TGFβ pathways converge during myofibroblast conversion. (**a**) NF#1 fibroblast matrix remodelling assay. NF#1 fibroblasts embedded in collagen gels were treated with BSA, Wnt7a or TGFβ1 in the presence or absence of SB43152 for 72 h. Recombinant Wnt7a was pre-absorbed with a neutralizing anti-TGFβ antibody before use (see Methods). Data show quantification of matrix remodelling by NF#1 fibroblasts±s.e.m. Groups were compared using two-way ANOVA followed by Bonferroni post-test. ***P<0.001. Equivalent results were obtained in two independent experiments. (**b**) Remodelled gels from **a** were fixed, sectioned and stained with antibodies against the pan-fibroblast marker Endo180 (*Mrc2*) (red) and phospho-Smad2 (green). Nuclei were counterstained with DAPI. Left panel, the mean fibroblast phospho-Smad2 scores±s.e.m. (*n*=3 remodelled gels in each group). One-way ANOVA with Bonferroni post-test was used to compare groups. **P<0.01. Right panel, representative images. Scale bar, 150 μm. (**c**) NF#1 fibroblasts embedded in collagen gels were treated with conditioned medium from 4T07 cells transfected with vector alone (4T07-Vec CM) or Wnt7a (4T07-Wnt7a CM) in the presence or absence of SB43152 (10 μM) for 72 h. Conditioned media were pre-absorbed with a neutralizing anti-TGFβ antibody before use (see Methods). Data show quantification of matrix remodelling±s.e.m. Two-way ANOVA with Bonferroni post-test was used to compare groups. ***P<0.001. (**d**,**e**) NF#1 or NIH-3T3 fibroblasts transfected with vector alone (−Vec) or Wnt7a (−Wnt7a) embedded in collagen gels were cultured in the presence or absence of SB43152 (10 μM). Data show quantification of matrix remodelling from five independent experiments±s.e.m. Groups were compared using two-way ANOVA. **P<0.01. Representative confocal images of NF#1 and NIH-3T3 gels stained with TRITC-phalloidin (red) and second-harmonic emission to image collagen density (green). Scale bar, 20 μm. (**f**) 4T1 and 4T07 tumour sections were stained for Endo180 (*Mrc2*) (red) and phospho-Smad2 (green). Nuclei were counterstained with DAPI. Left panel, representative images taken from the tumour–stroma interface. Arrowheads indicate Endo180-positive fibroblasts with high nuclear phospho-Smad2. Scale bar, 50 μm. Right panel, the mean fibroblast phospho-Smad2 scores (*n*=5 tumours in each group). Comparison was made using Student's *t*-test.

To date, the pathway most frequently implicated in extracellular matrix mechanosensing and myofibroblast differentiation is TGFβ signalling^2^,^34^,^35^,^36^. Therefore, we investigated next whether there is an interplay between Wnt7a and TGFβ signalling during fibroblast activation. In classical TGFβ signalling, TGFβ binding to a target cell results in phosphorylation of Smad2 and Smad3 and their translocation into the nucleus where they trigger transcriptional activation of target genes including αSMA. Indeed, in both NF♯1 (Fig. 5a,b) and NIH-3T3 (Supplementary Fig. 5a,b) fibroblasts plated in 3D collagen gels, the Wnt7a-dependent increase in fibroblast contractility is accompanied by efficient nuclear translocation of phosphorylated Smad2. Moreover, treatment with the TGFβ type I receptor (TβR1; ALK5) inhibitor SB431542 not only effectively blocked Wnt7a-mediated phospho-Smad2 nuclear translocation but also Wnt7a-mediated fibroblast contraction (Fig. 5a,b and Supplementary Fig. 5a,b). Further, ALK5 inhibition also effectively blocked the enhanced fibroblast contractility in 3D collagen gels following treatment with conditioned medium from 4T07 cells ectopically expressing Wnt7a (Fig. 5c). Similarly, the enhanced contractile force that NF♯1 (Fig. 5d) and NIH-3T3 (Fig. 5e) fibroblasts ectopically expressing Wnt7a exert on the extracellular matrix, as monitored by 3D collagen gel contraction (left panels) and a locally increased density of collagen fibrils (right panels), was completely blocked by the ALK5 inhibitor SB431542.

These data support a model in which Wnt7a, in a manner that is dependent on malleability of the mechanical substrate, potentiates TGFβ receptor signalling and that this potentiation of TGFβ receptor activity is critical for myofibroblast conversion. In support of this model, fibroblasts associated with 4T1 tumours show a significantly increased TGFβ pathway activation compared with 4T07 tumour fibroblasts as monitored by higher phospho-Smad2 levels within the fibroblast nuclei (Fig. 5f).

### Wnt7a-activated CAFs promote invasion and metastasis

**Figure 6:**
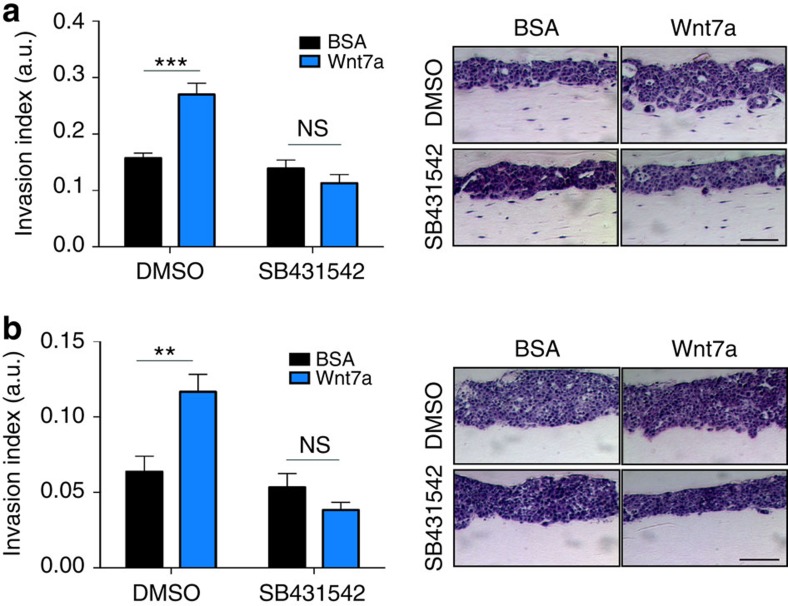
Wnt7a-driven myofibroblast conversion increases tumour cell invasion. (**a**) Organotypic assays of breast cancer cell invasion into NF#1 fibroblast-containing matrix. Left panel: quantification of invasion index (a.u.) by measuring the total area over which cancer cells had dispersed (including invading and non-invading cells) and the area of non-invading cells. The value shown is the average 1—(non-invading area/total area) of at least 13 measurements±s.e.m. Right panel, representative images used for invasion index measurements. Scale bar, 50 μm. Two-way ANOVA with Bonferroni post-test used to compare groups. ***P<0.001. Equivalent results were obtained in two independent experiments. (**b**) Organotypic assays of cell invasion into a NF#1 fibroblast-conditioned matrix. Left panel: quantification of invasion index (a.u.) and statistical analysis as in **a** for seven measurements per gel±s.e.m. Right panel: representative images used for invasion index measurements. Scale bar, 50 μm. Two-way ANOVA with Bonferroni post-test used to compare groups. **P<0.01. Equivalent results were obtained in two independent experiments.

In light of our findings that Wnt7a promotes an altered stromal compartment in aggressive tumours, we next characterized the functional properties of Wnt7a-activated CAFs. In organotypic assays in which tumour cells are plated on a 3D matrix containing live fibroblasts, Wnt7a treatment significantly enhances tumour cell invasion (Fig. 6a). Importantly, in a complementary approach where Wnt7a-treated fibroblasts are allowed to remodel the 3D matrix but are killed before tumour cell invasion is assessed, we demonstrate that the increased tumour cell invasion is dependent on the ability of the treated fibroblasts to remodel the matrix (Fig. 6b). Importantly, the ability of Wnt7a-activated fibroblasts to promote tumour cell invasion is effectively blocked by the ALK5 inhibitor SB421542 (Fig. 6), further supporting a mechanism in which Wnt7a promotes the functional differentiation of fibroblasts into activated CAFs in a TGFβ signalling-dependent manner.

**Figure 7:**
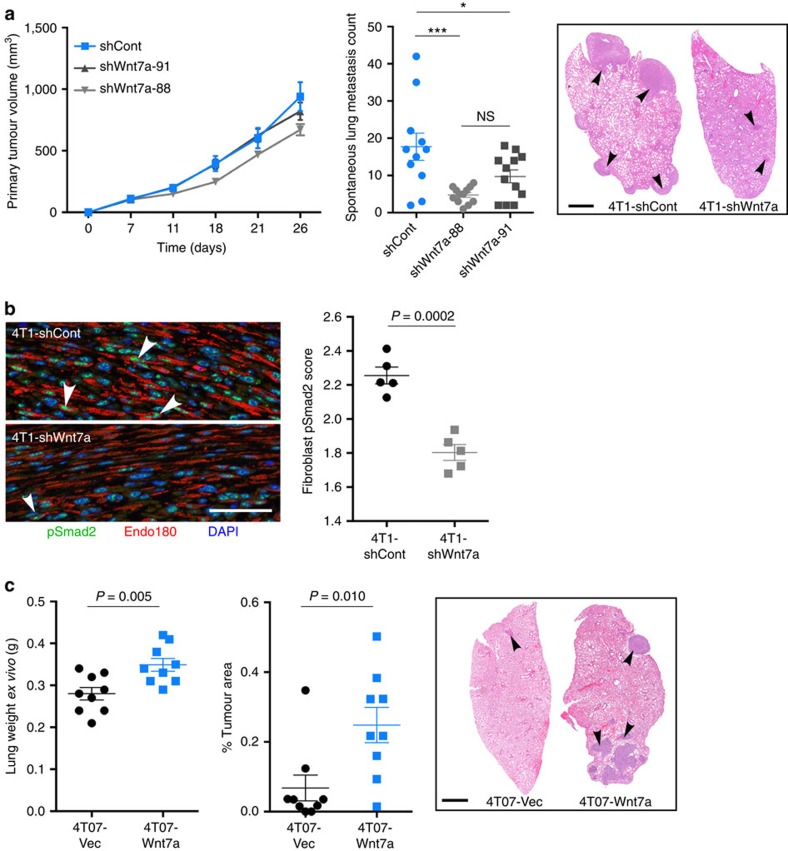
Wnt7a promotes tumour metastasis and activates the TGFβ pathway in fibroblasts. (**a**) In all, 1 × 10^4^ 4T1 cells infected with control (4T1-shCont) or two independent Wnt7a (4T1-shWnt7a-91 and 4T1-sh-Wnt7a-88) shRNAs (see Supplementary Fig. 6a for qPCR analysis) were inoculated into Balb/c mice, *n*=11–12 mice per group. Primary tumour volume was measured until tumours reached maximum size (day 26), *P*=NS (nonsignificant). One-way ANOVA with Bonferroni post-test used to compare groups. *P<0.05; ***P<0.001. Metastasis to the lung was quantified as the number of tumour nodules from sections taken mid-way through the lung. Representative histology sections of metastases in the lungs are shown. Arrowheads indicate tumour nodules. Scale bar, 1 mm. (**b**) 4T1-shCont and 4T1-shWnt7a primary tumours were fixed, paraffin-embedded, and phospho-Smad2 nuclear localization in fibroblasts at the tumour–stroma interface was quantified as in Fig. 5b. Left panel, representative images taken at the tumour–stroma interface. Arrowheads indicate Endo180-positive fibroblasts with high nuclear phospho-Smad2. Right panel, data shown are the mean fibroblast phospho-Smad2 scores per tumour (*n*=5 tumours in each group). (**c**) In all, 2.5 × 10^5^ 4T07-Vec or 4T07-Wnt7a cells were inoculated into the tail vein of Balb/c mice. Mice were killed on day 19 and tumour burden in the lung was assessed by lung weight and quantification of % tumour area from sections taken mid-way through the lung. Data shown are from nine mice per group±s.e.m. Comparison was made using Student's *t*-test. Representative histology images are shown. Arrowheads indicate tumour nodules. Scale bar, 1 mm.

Two complementary approaches were taken to investigate the role of Wnt7a in promoting tumour aggressiveness of *in vivo* breast cancer models. First, the effect of short hairpin RNA (shRNA)-mediated downregulation of Wnt7a expression was assessed in a spontaneous metastasis assay. Targeting Wnt7a with either of two independent shRNAs (Supplementary Fig. 6a) has no effect on primary tumour growth (Fig. 7a) but results in an overall trend to decreased αSMA and fibroblast activation protein (FAP) expression by tumour fibroblasts (Supplementary Fig. 6b). Importantly, this impairment in CAF activation correlates with a significant reduction in spontaneous metastasis to the lung (Fig. 7a). In addition, Wnt7a downregulation significantly impairs TGFβ pathway activation in the CAFs as monitored by the reduced levels of phospho-Smad2 detected within fibroblast nuclei (Fig. 7b), demonstrating that the same signalling pathways are active both *in vitro* and *in vivo.* Second, in an experimental metastasis assay, ectopic expression of Wnt7a in the less aggressive 4T07 cells significantly enhances metastatic lung colonization as monitored by *ex vivo* lung weight and histological quantification (Fig. 7c).

### Wnt7a correlates with stromal dysplasia and poor prognosis

**Figure 8:**
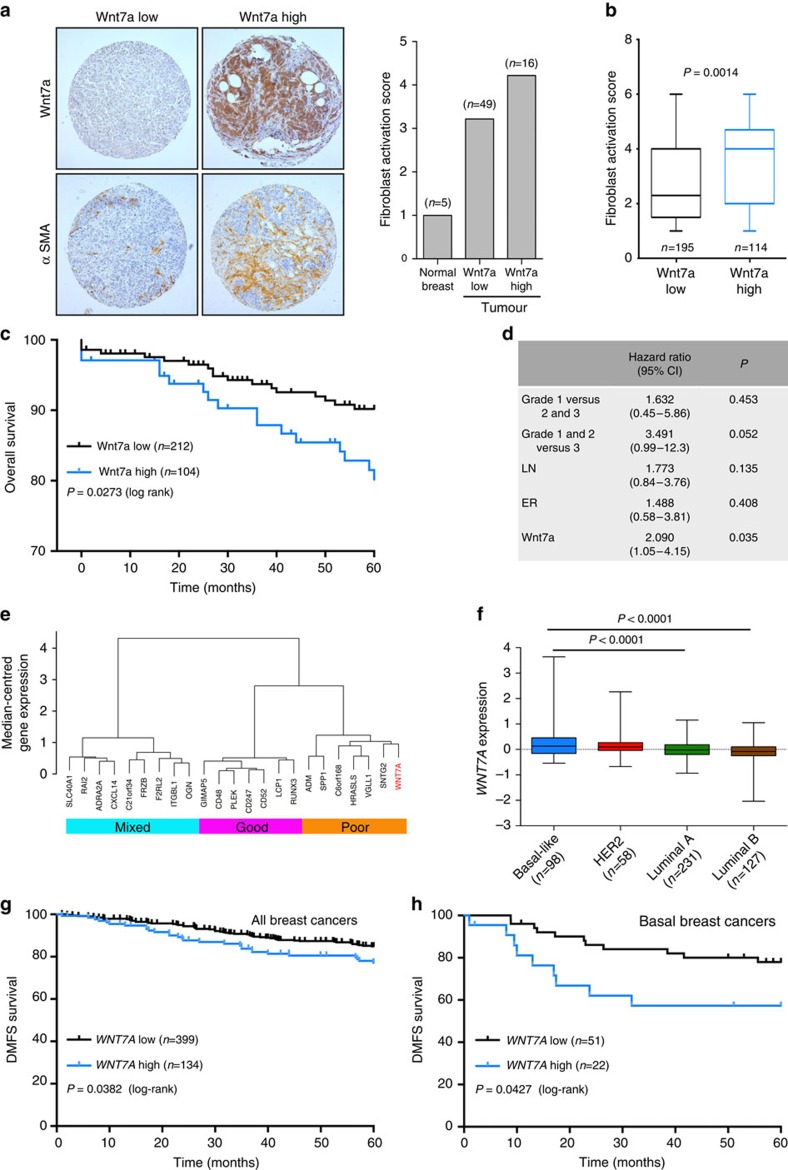
Tumour Wnt7a expression correlates with breast cancer stromal activation and patient outcome. (**a**) A tissue microarray comprising 65 scoreable invasive breast cancers and 5 normal breast samples was assessed for Wnt7a and αSMA expression using immunohistochemistry. Shown are representative 0.6-mm cores with low or high tumour cell Wnt7a expression, and the matched αSMA-stained cores. Graph shows correlation of Wnt7a expression with the ‘fibroblast activation score' (see Methods section). (**b**) Assessment of Wnt7a protein expression and fibroblast activation in an independent (Leeds) tissue microarray comprising 347 scoreable breast tumours using the same scoring criteria as in **a**. (**c**) Kaplan–Meier curve showing Wnt7a high protein expression in the Leeds samples was significantly associated with decreased overall survival (*P*=0.0273, log-rank Mantel–Cox). (**d**) Cox multivariate analysis showing that Wnt7a expression in the Leeds samples is a significant predictor of overall survival independent of oestrogen receptor (ER), grade and lymph node (LN) status. Hazard ratios with 95% confidence intervals (CIs) are shown. (**e**) Correlation of *WNT7A* expression with the Finak SDPP. Unsupervised hierarchical clustering of the SDPP genes and *WNT7A* was performed with the 522 primary breast cancers available in the TCGA data set. *WNT7A* was clustered with the poor-prognosis SDPP genes. Pearson correlation showed a significant correlation between *WNT7A* expression with centroid gene expression of the SDPP poor-prognosis group (*r*=0.213, *P*<0.0001). (**f**) *WNT7A* expression was examined in the TCGA data set of breast cancers (*n*=514). Using one-way ANOVA and Tukey's multiple comparison test, high *WNT7A* gene expression was significantly associated with the basal-like intrinsic subtype (*P*<0.0001). (**g**,**h**) Kaplan–Meier curves showing high *WNT7A* expression in 533 systemically untreated breast cancers^57^, and the 73 systemically untreated basal breast cancers is significantly associated with poor distant metastasis-free survival (*P*=0.0382 and *P*=0.0427, log-rank Mantel–Cox, respectively).

Consistent with our findings in mouse mammary tumours (Fig. 2d), in human breast cancers Wnt7a protein expression is predominantly associated with the tumour cell compartment, with lower levels detectable within the stroma (Supplementary Fig. 7). To address the clinical significance of Wnt7a expression in tumours, we first examined whether Wnt7a expression in human breast cancer correlates with a desmoplastic stroma. Immunohistochemical staining of an initial set of 65 human invasive breast cancers revealed an increased CAF activation in tumours with high Wnt7a expression (Fig. 8a). These findings were then extended to a larger, independent cohort of 347 breast cancers^11^. Consistent with the initial set, tumours expressing high levels of Wnt7a have a significantly increased desmoplastic response (Fig. 8b), characterized by more numerous αSMA-positive CAFs (*P*=0.0576) as well as increased αSMA levels in the recruited CAFs (*P*=0.0009; Supplementary Fig. 8a). Second, taking a complementary approach, we examined the fibroblast core serum response (F-CSR) signature described in ref. 37 as a surrogate measure of CAF activation in clinical data sets, and demonstrated a significant correlation between high *WNT7A* expression in human breast cancers and a high F-CSR score (*P*=2.56 × 10^−5^; Supplementary Fig. 8b). In addition, in agreement with our *in vitro* and *in vivo* data, using the fibroblast TGFβ response signature (F-TBRS) described in ref. 38, we also show a significant correlation between high *WNT7A* expression and a high F-TBRS score (*P*=0.005; Supplementary Fig. 8c), further supporting the convergence of Wnt7a and TGFβ signalling pathways to drive fibroblast activation.

To examine the clinical significance of these findings we used three different approaches. First, examination of levels of Wnt7a protein expression in the 347 breast cancer cohort (Fig. 8b) revealed that overall patient survival is significantly reduced in the Wnt7a high versus the Wnt7a low group (*P*=0.0273 log-rank test; Fig. 8c), and that, by multivariate analysis, Wnt7a expression is an independent prognostic outcome marker (Fig. 8d). Second, we performed unsupervised hierarchical clustering of the Finak *et al*.^39^ stroma-derived prognostic predictor (SDPP) genes and *WNT7A* in The Cancer Genome Atlas (TCGA) primary breast cancer data set and demonstrated that *WNT7A* clusters with the SDPP poor prognostic genes (Fig. 8e). Further, Pearson correlation shows that there is a significant association between *WNT7A* gene expression and the gene expression centroid of the SDPP poor prognostic group (*r*=0.213, *P*<0.0001). Finally, examination of *WNT7A* gene expression in the TCGA breast cancer data set demonstrated an increased Wnt7a expression in primary tumours versus matched normal tissue (paired *t*-test *P*=0.0098) and that within primary tumours high *WNT7A* gene expression is significantly associated with basal-like breast cancer (one-way analysis of variance (ANOVA), *P*<0.0001; Fig. 8f). High *WNT7A* expression is also associated with ER− versus ER+ tumours (unpaired *t*-test for unequal s.d. with Welch's correction, *P*=0.0002), triple-negative versus non-triple-negative tumours (unpaired *t*-test for unequal s.d. with Welch's correction, *P*=0.0031) and primary tumours versus matched normal tissue (*n*=22 pairs; paired *t*-test, *P*=0.0013). Importantly, consistent with the tissue microarray (TMA) analysis presented here, there is a significant correlation between high *WNT7A* gene expression and poor outcome (distant metastasis-free survival) in breast cancer patients, either when examining all systemically untreated breast cancers (log-rank *P*=0.0382; Fig. 8g) or only the basal breast cancers (log-rank, *P*=0.0427; Fig. 8h).

## Discussion

The main aim in this study was to identify the mechanisms underlying the striking heterogeneity in stromal composition between different breast tumours. We demonstrate that the 4T1 tumour cell line series recapitulates human disease in showing a strong correlation between a desmoplastic response and tumour aggressiveness, and making it a valid model for studying heterogeneity of the CAF compartment *in vivo*. While others^40^,^41^,^42^ have performed gene expression profiling of 4T1 series tumours, our study is the first to interrogate the purified tumour cell and fibroblast populations separately and to overlay the data to uncover previously unknown players in the reciprocal signalling between tumour cells and their associated CAFs. Using this model we identified Wnt7a as a novel factor secreted by aggressive tumour cells that drives the acquisition of a desmoplastic response characterized by an activated CAF phenotype, which is capable of matrix remodelling and promotion of tumour cell invasion. Our findings also support a role for Wnt7a in the recruitment of a desmoplastic stroma in human tumours, whereby high Wnt7a expression is associated with enhanced CAF activation, TGFβ pathway activation within the CAF compartment and expression of poor-prognosis stromal genes as defined by the Finak *et al*.^39^ SDPP. Most importantly, high Wnt7a expression is associated with reduced overall and metastasis-free breast cancer patient survival. While this is the first report of Wnt7a as a prognostic marker in breast cancer, Wnt7a has been documented to be upregulated in malignant as compared with borderline or benign ovarian tumours and to promote tumour growth in *in vivo* ovarian cancer models^30^.

In interrogating the mechanism by which Wnt7a promotes fibroblast activation, our studies have provided evidence that implicates Wnt7a as a modulator of TGFβ signalling. The convergence of TGFβ and Wnt signalling in both development and disease has been documented previously; however, the intricacies of this interaction are complex and clearly dependent on the particular TGFβ and Wnt family ligand as well as cell and tissue context^43^. To date, the majority of reports have focused on the convergence of Wnt and TGFβ signalling at the level of their respective downstream pathways, for example, the association of Smads and LEF-1 or, more indirectly, by Wnt-mediated transcriptional upregulation of TGFβ family ligands and *vice versa*^44^. Recent studies in mesenchymal cells have attributed the signalling interaction between Wnt5a and TGFβ to the activation of Wnt-mediated Ca2+ and JNK, but not β-catenin, pathways^45^, while others have reported that β-catenin-dependent Wnt signalling is necessary for TGFβ-mediated fibrosis^46^. In our study, we find no evidence that Wnt7a promotes β-catenin signalling or activates the CaMKII or planar cell polarity pathways in fibroblasts. Rather, our data support a model in which a complete myofibroblast conversion requires Wnt7a-mediated potentiation of TGFβ receptor activity and we suggest, less traditionally, that this occurs at the level of the TGFβ receptor rather than via downstream signalling components. Interestingly, it has recently been reported that Wnt5a expression is required to potentiate TGFβ signalling during colonic epithelial wound repair and that, in a similar manner to that reported here, these effects were dependent on TβR1 kinase activity, as evidenced by their blockade following ALK5 inhibition^26^. The authors have proposed that this interaction may provide a mechanism for Wnt5a to regulate the localization and timing of TGFβ signalling during stem cell proliferation^47^. Such a model is consistent with our observation that 4T07 and 4T1/410.4 tumour cells isolated from *in vivo* orthotopic tumours did not differ in their TGFβ expression, yet the tumours displayed striking differences in stromal composition, which is characterized by TGFβ signalling activation. Therefore, Wnt7a may be regulating the intricacies of the location and timing of TGFβ signalling. Indeed, FACSorted CAFs from Wnt7a high 410.4/4T1 tumours consistently show higher levels of *Tgfb1* expression as compared with Wnt7a low 4T07 tumours (Fig. 1c). TGFβ is well known to have the capacity to act in an autocrine manner, and it is therefore tempting to speculate that Wnt7a could lead, over the longer term, to the establishment of a self-sustaining autocrine TGFβ signalling loop that maintains CAF activation.

As the regulatory mechanisms of TGFβ signalling are extremely complex^48^ and context-dependent, the molecular nature of TβRI activation could be manifold. The potentiation of TβRI kinase signalling could be mediated directly by the interaction with a Wnt7a receptor component. For example, it has recently been reported that Wnt ligands bound to the LRP5/6 co-receptors physically interact with both TβR1 and PDGFRβ in mesenchymal cells^49^ and that the Ror2 co-receptor is responsible for Wnt5a-mediated activation of TGFβ signalling in colonic epithelial stem cells^26^. Alternatively, Wnt7a could interact indirectly with TβR1 via its association with other transmembrane receptors. For example, Wnt7a bound to Frizzled-7 has been demonstrated to associate with syndecan-4 and that binding of this receptor complex to fibronectin is required to drive symmetric expansion of satellite stem cells in regenerative myogenesis^27^. Although there are no reports of syndecan-4 binding to TβR1, syndecan-4 can trap TGFβ1 on the cell surface^50^, bringing it closer to its receptor and thus providing a potential indirect mechanism linking Wnt7a to TβR1. It is tempting to speculate that, if the same pathways are active in fibroblasts, this would lead to an autocrine signalling loop that stabilizes the fibroblast activation state within tumours. In conclusion, our findings highlight a novel level of interaction between Wnt and TGFβ pathways—two of the key signalling players in both development and disease. Disruption of this interaction may be a promising strategy to disrupt tumour:stroma crosstalk to prevent CAF recruitment or to break autocrine CAF signalling loops, thereby reversing the production of a pro-tumorigenic activated stroma.

## Methods

### Antibodies

Sources and dilutions used for the following antibodies are as follows. αSMA (clone 1A4, A5228, Sigma; 1:2,000 for immunoblotting (IB), 1:500 for immunohistochemistry), αSMA-fluorescein isothiocyanate (FITC; clone 1A4; F3777, Sigma; 1:1,000 for immunofluorescence (IF)), phospho-CaMKII (Thr286; D21E4, 12716, Cell Signaling, 1:1,000), β-catenin (14, BD Biosciences, 1:500 for IB), phospho-β-catenin (9566, Cell Signaling, 1:1,000 for IB), CD45-PE-Cy5 (30-F11, BD Pharmingen, 1:100 for FACS), endomucin (V.7C7, Santa Cruz Biotechnology, 1:1,000 for IF), endosialin (P13, see (ref. 51), 1:2,000 for IF), Endo180 (*Mrc2*; AF4789, R&D Systems, 1:100 for IF), FAP (ab28244, Abcam, 1:100 for IF), c-Jun (60A8, 9165, Cell Signaling, 1:1,000 for IB), phospho-c-Jun (54B3, Cell Signaling, 1:1,000 for IB), TGFβ (1D11 neutralizing antibody, R&D Systems), SAPK/JNK (9252, Cell Signaling, 1:1,000 for IB), phospho-SAPK/JNK (9251, Cell Signaling, 1:1,000 for IB), phospho-Smad2 Ser465/467 (3101, Cell Signaling, 1:100 IF), α-tubulin (B-5-1-2, Sigma, 1:25,000 for IB), vinculin (E1E9V, 13901, Cell Signalling, 1:2,000 for IB), Wnt7a (ab100792, Abcam, 1:200 for immunohistochemistry), Alexa-conjugated secondary antibodies (Invitrogen, 1:1,000 dilution), horseradish peroxidase (HRP)-conjugated secondary antibodies (Jackson ImmunoResearch 1:5,000 for IB or Santa Cruz Biotechnology 1:10,000 for IB). TRITC-phalloidin (Sigma P1951, 1:500 for IF). Uncropped immunoblots are shown in Supplementary Fig. 9.

### Reagents

Recombinant growth factors were as follows: BMP-7, CXCL16, Dhh, GM-CSF, PDGF-BB, TGFβ1, Wnt3a and Wnt7a (R&D Systems). SB431542 TGFβR1 inhibitor (10 μM) and SB216763 GSK-3β inhibitor (10 μM) are both from Tocris; fibronectin, human plasma are from (341635) Calbiochem and Protein G Dynabeads (10003D) from Life Technologies).

### Gene expression profiling of cell populations from tumours

All animal work was carried out with UK Home Office approval. Overall, 5 × 10^5^ 4T1, 410.4 or 4T07 tumour cells suspended in 50 μl of a 1:1 mixture of PBS and growth factor-reduced Matrigel were injected into the fourth mammary fat pad of 6–8-week-old female wild-type Balb/c or Ub-GFP Balb/c mice (Balb/c mice expressing GFP under the human ubiquitin C promoter)^52^.

For isolation of tumour cells and fibroblasts, primary tumours in Ub-GFP Balb/c mice were removed at an average diameter of 10 mm, homogenized using a McIlwain Tissue Chopper (Campden Instruments) and digested in L-15 medium containing 3 mg ml^−1^ collagenase type I at 37 °C for 1 h, followed by digestion with 0.025 mg ml^−1^ DNase at 37 °C for 5 min. After erythrocyte lysis using Red Blood Cell Lysing Buffer (Sigma), single cells were resuspended at 1–2 × 10^7^ cells per ml in PBS and stained with CD45-PE-Cy5 and 4,6-diamidino-2-phenylindole for FACSorting using a FACSAria flow cytometer (BD Biosciences). Sorted cells were pelleted and directly lysed in TRIzol (Invitrogen) for RNA extraction. RNA was analysed using the RNA 6000 Nano kit on a 2100 BioAnalyser (Agilent), and only samples with an RNA integrity number >9 were used for further experiments.

For each tumour cell and fibroblast population, 100 ng of RNA from three independent biological replicates was amplified using the TotalPrep 96-RNA amplification kit from Ambion (Applied Biosystems). cRNA (1.6 μg) was hybridized to the MouseWG-6 v2.0 Expression BeadChip (Illumina). Expression data were analysed in R (www.bioconductor.org), using the *lumi* package for probe selection. Data were transformed using variance stabilization and normalized using quantile normalization. Pairwise comparisons were carried out using the *limma* package. False discovery rate was used to correct for multiple testing. When different sample groups were pooled for pairwise comparison, the replicates of the different groups were considered replicates of a single group. Data for heatmap presentation were prepared in Cluster 3.0, and the heatmaps were generated using Java Treeview. Database for Annotation, Visualization and Integrated Discovery (DAVID) v6.7 (National Institute of Allergy and Infectious Diseases) was used for functional annotation analyses. Microarray data have been deposited in NCBI Gene Expression Omnibus (GSE50471).

### Cell culture and assays

ZR75.1 cells were obtained from American Type Culture Collection and short tandem repeat tested every 4 months using the StemElite ID System (Promega). Generation of the NF#1 fibroblasts (immortalized normal mouse mammary fibroblasts) have been described previously^22^. In brief, fibroblasts from 8–10-week-old female FVB/n mice were cultured in DMEM plus 10% FCS (fetal calf serum) and 1% ITS (insulin–transferrin–selenium; Invitrogen) supplement and media changed daily. After expansion in culture, fibroblasts were immortalized by infection with HPV-E6 retrovirus and selected using 2.5 μg ml^−1^ puromycin.

NIH-3T3 fibroblasts were from Isacke Laboratory stocks. 4T1 series cell lines were Sahai Laboratory stocks. Cells were maintained in DMEM plus 10% FCS in tissue culture-treated plastic or on fibronectin-coated Softwell hydrogels (50 kPa). NF#1 cells (immortalized normal mouse mammary fibroblasts)^22^ were additionally supplemented with 1% ITS. Cells ectopically expressing mouse Wnt7a were generated by infection with pHIV-Wnt7a-RFP lentivirus. Briefly, pDEST/pHIV-H2BmRFP-rfa_verB-Wnt7a vector plasmid, packaging plasmid psPAX2 and envelope plasmid pMD2.G were co-transfected into HEK293T cells using Lipofectamine 2000. Virus particles were collected and used to infect the cells. RFP-expressing cells were selected by FACSorting. To establish stable knockdown of Wnt7a in 4T1 cells, cells were infected with Mission particles (Sigma) containing one of two independent Wnt7a shRNA (shWnt7a-88, TRCN0000071788 and shWnt7a-91, TRCN0000071791) or control shRNA (shCont; SHC001V) at an multiplicity of infection of 2. Infected cells were selected using puromycin (Sigma) at 2.5 μg ml^−1^ for 5–7 days.

For *in vitro* assays, cells were starved overnight in serum-free Advanced DMEM (Invitrogen) before growth factor addition. Where indicated, cells were plated on fibronectin-coated Softwell hydrogels (50 kPa) in serum-free Advanced DMEM.

For TOPflash assay, cells were transiently co-transfected with pCMV-Green-Renilla and the TOPflash and FOPflash plasmids and, where indicated, the delta-GSK plasmid was also transiently co-transfected in the mixture. Twenty-four hours post transfection, cells were starved in serum-free Advanced DMEM overnight and then treated with bovine serum albumin (BSA), Wnt7a or SB216763 for 24 h before measuring luciferase activity with the Dual-Glo Luciferase Assay System (Promega).

For conditioned medium production, cells at ∼70% confluency were washed in serum-free medium and then incubated in serum-free medium for 48 h. Conditioned medium was filtered (0.45 μm) before use.

For cell viability assays, cells were plated at 5 × 10^2^ cells per well in 96-well plates. The following day, the medium was replaced with DMEM plus 2% FCS. Six hours later, BSA or Wnt7a (100 ng ml^−1^) was added (day 0). At the indicated time points, medium was aspirated and cell number was measured by adding 200 μl CellTiter-Glo.

For matrix remodelling assays, 7.5 × 10^4^ fibroblasts were embedded in 100 μl of 1:1 mixture of collagen I and growth factor-reduced Matrigel (BD Biosciences), yielding final concentrations of ∼4.6 and ∼2.2 mg ml^−1^, respectively, and seeded into 24-well glass bottom MatTek plates. Once the gel was set, cells were maintained in DMEM plus 1% FCS, 1% ITS containing BSA, Wnt7a (100 ng ml^−1^) or TGFβ1 (1 ng ml^−1^) in the presence or absence of SB431542 (10 μM) for 72 h. To obtain a contraction value, the gel area was measured using the Image J software and fold contraction over control gels (BSA-treated or control vector-transfected) was calculated. Where indicated, gels were then formalin-fixed and paraffin-embedded. Sections were stained for Endo180 (*Mrc2*) and phospho-Smad2 as described below, and 6–10 confocal images per gel were collected. Where indicated, Wnt7a or conditioned media were pre-absorbed with a neutralizing anti-TGFβ antibody before use as follows. Neutralizing anti-TGFβ antibody (1D11, R&D Systems) was incubated with Protein G Dynabeads for 15 min at room temperature with rotation. Beads were washed twice and incubated with recombinant Wnt7a (100 ng ml^−1^) or conditioned media for 1 h at room temperature with rotation. Beads were removed by magnetic separation and the supernatant added to the contraction gel assays.

Organotypic invasion assays were undertaken as described previously^22^. In brief, 1 × 10^6^ fibroblasts were embedded in a mixture of collagen I/growth factor-reduced Matrigel matrix. After the gel was set at 37 °C for 1 h, DMEM plus 10% FCS and 1% ITS was added. Sixteen hours later, 5 × 10^5^ 4T1 cells were seeded on top in DMEM plus 10% FCS and 1% ITS. After 8 h, a thin layer of gel was added covering the tumour cells. The gel was then mounted on a metal bridge and fed from underneath with DMEM plus 10% FCS and 1% ITS (changed daily). After 6 days, the cultures were fixed using 4% paraformaldehyde plus 0.25% glutaraldehyde in PBS and processed by standard methods for haematoxylin and eosin staining. For assays involving the removal of fibroblasts (fibroblast-conditioned matrix), the fibroblasts were left to remodel the gel for 5 days, after which the gels were incubated in DMEM plus 10% FCS and 1% ITS plus hygromycin (400 μg ml^−1^) for 48 h to kill the fibroblasts and then washed three times with DMEM plus 10% FCS and 1% ITS (>30 min per wash). Then, 5 × 10^5^ 4T1 cells were plated on top and the assays proceeded as usual.

### qPCR

Quantitative real-time RT–PCR (qRT–PCR) was performed using 5–12.5 ng cDNA per well with 0.5 μl Taqman Gene Expression Assay probe and 5 μl qPCR MasterMix in a 10-μl reaction volume on an ABI Prism 7900HT sequence detection system. Each reaction was performed in triplicate. Data analysis was performed using the SDS 2.2.1 software (Applied Biosystems). Fold change was determined in relative quantification units using *B2m*/*B2M* for normalization.

### *In vivo* assays

For growth factor treatment, the 4T07 tumour cell suspension was supplemented with recombinant growth factors before inoculation of 5 × 10^5^ tumour cells suspended in 50 μl of a 1:1 mixture of PBS and growth factor-reduced Matrigel bilaterally into the fourth mammary fat pad of 6–8-week-old female wild-type Balb/c mice. Growth factors were injected three times per week in 50 μl of PBS into the fourth mammary fat pad, or directly into the centre of the tumour when tumours were palpable. Concentrations of recombinant proteins were as follows: BMP-7, 100 ng ml^−1^; CXCL16, 500 ng ml^−1^; Dhh, 1 μg ml^−1^; GM-CSF, 50 ng ml^−1^; PDGF-BB, 300 ng ml^−1^; Wnt7a, 100 ng ml^−1^; and TGFβ1, 1 ng ml^−1^. Tumour volume was monitored from day 7 onwards. The experiment was terminated when tumour size of the first mouse reached the maximum average diameter of 12 mm.

Overall, 3 × 10^6^ 168FARN-Wnt7a or 168FARN-Vec cells were suspended in 100 μl of a 1:1 mixture of PBS and growth factor-reduced Matrigel and inoculated into 6–8-week-old female Balb/c mice. Overall, 2 × 10^6^ ZR75.1-Wnt7a or ZR75.1-Vec cells were suspended in 100 μl of PBS and inoculated into 6–8-week-old female CB17 nonobese diabetic severe combined immunodeficient mice. For experimental metastasis, 2.5 × 10^5^ 4T07-Wnt7a or 4T07-Vec cells were injected into the tail vein of 6–8-week-old female Balb/c mice. For spontaneous metastasis, 1 × 10^4^ 4T1-shWnt7a or 4T1-shCONT cells were inoculated into the fourth mammary fat pad of 6–8-week-old female Balb/c mice. Tumour volumes were measured until they reached maximum allowable size when the animals were killed (day 26) and the lungs removed at necroscopy.

### Immunostaining of mouse tumours and matrix remodelling gels

Formalin-fixed, paraffin-embedded tumour or matrix remodelling gel sections were stained with antibodies against endosialin^51^, αSMA and endomucin, antibodies against the pan-fibroblast marker Endo180 (Mrc2)^53^,^54^ and phospho-Smad2 or antibodies against FAP and αSMA and were imaged with confocal laser scanning microscopy^55^. Phospho-Smad2 staining in the fibroblast (Endo180-positive) nuclei was scored as 1 (negative), 2 (low) and 3 (strong). Alternatively, sections were subjected to αSMA immunohistochemistry and HRP staining was quantified using Image J.

### Clinical material

The Breakthrough TMA has been previously described^56^. In brief, following ethical approval (RMH REC), the TMA was constructed with replicate 0.6-mm cores of invasive breast carcinomas from patients diagnosed and treated at the Royal Marsden NHS Foundation Trust between 1994 and 2000. The Leeds TMA including patient characteristics has been previously described^11^. In brief, following ethical approval (Leeds (East) REC:06/Q1206/180), the TMA was assembled with triplicate cores from 358 cases of breast cancer diagnosed at the Leeds Teaching Hospitals NHS Trust between 1987 and 2005. TMAs were stained with anti-αSMA (clone 1A4, 1:500 dilution) or anti-Wnt7a (1:200 dilution) followed by HRP-conjugated secondary antibodies. Bound antibodies were visualized using diaminobenzidine (EnVision, Dako) before counterstaining with haematoxylin. All TMA scoring was performed blind. Wnt7a protein expression in the tumour cells was scored as negative/low or high. Fibroblast activation score=(fibroblast abundance × fibroblast αSMA expression) where fibroblast abundance was scored as 1 (few fibroblasts) or 2 (abundant fibroblasts) and fibroblast αSMA expression was scored as 1 (none/little), 2 (intermediate) or 3 (strong). Scores across the triplicate cores were averaged.

### Statistical and bioinformatics analyses

The Graphpad Prism version 6.0c statistical package was used for statistical analysis, except for multivariate analysis of clinical data where SPSS was used. Numerical data are expressed as mean±s.e.m. Experiments were analysed with unpaired Student's *t*-test. All tests were two-tailed, with a confidence interval of 95%. Where more than two groups were compared, one-way ANOVA followed by Bonferroni post-test with confidence interval of 95% was used. Where more than one group with or without inhibitor or tumour growth curves were compared, two-way ANOVA followed by Bonferroni post-test with confidence interval of 95% was used. *P<0.05; **P<0.01; ***P<0.001.

Bioinformatic analysis of gene expression profiling data is described above. Clinical relevance of variable *WNT7A* expression was assessed using publicly available data from ref. 57. For survival analysis, the highest quartile of gene expression was used to dichotomize the 533 systemically untreated breast tumours into high and low groups.

For correlation of Wnt7a expression with the Chang *et al*.^37^ F-CSR signature, the F-CSR signature was retrieved from http://changlab.stanford.edu/2005-PNAS-Data.html. In the NKI295 data set, 442 probes representing 380 out of the 459 F-CSR genes were present, of which 212 probes were serum-induced with centroid values >0, and 230 probes were serum-repressed with centroid values <0. Gene expression of the 442 probes from NKI295 data set was used to calculate a module score for each sample. The module scored is a weighted averaging^58^ (Supplementary Fig. 8b).

For correlation of Wnt7a expression with the Calon *et al*.^38^ F-TBRS, the F-TBRS signature was retrieved from http://dx.doi.org/10.1016/j.ccr.2012.08.013. There were 286 probes representing 171 unique annotated genes. In the TCGA data set 151 out of the 171 F-TBRS genes were matched. Average gene expressions of the 151 genes from TCGA data set was used as the signature score for each tumour. *WNT7A* gene expression was significantly correlated with F-TBRS signature (*r*=0.123, *P*=0.005) using Pearson correlation in the 522 primary breast cancer of the TCGA data set for which gene expression data were available (http://tcga-data.nci.nih.gov/docs/publications/brca_2012/BRCA.exp.547.med.txt).

For correlation of Wnt7a expression with the Finak *et al*.^39^ SDPP, the SDPP was retrieved from GeneSigDB (http://compbio.dfci.harvard.edu/genesigdb/). The SDPP contains 26 genes, of which 10 are associated with good patient prognosis, 10 with mixed prognosis and 6 with poor prognosis. In the TCGA data set of 522 primary breast cancers, unsupervised hierarchical clustering (Pearson correlation distance, Ward cluster method) was performed using the mapped median-centred gene expression. *WNT7A* clustered together with the SDPP poor-prognosis genes (Fig. 8e). Pearson correlation resulted in a significant association between *WNT7A* gene expressions with centroid values of poor-prognosis genes (*r*=0.213, adjusted *P*<0.0001).

## Additional information

**How to cite this article:** Avgustinova, A. *et al*. Tumour cell-derived Wnt7a recruits and activates fibroblasts to promote tumour aggressiveness. *Nat. Commun.* 7:10305 doi: 10.1038/ncomms10305 (2016).

## Supplementary Material

Supplementary Information Supplementary Figures 1-9, Supplementary Table 1 and Supplementary References
