# Supplementary material for: Tumour cell-derived Wnt7a recruits and activates fibroblasts to promote tumour aggressiveness
Source: Nat Commun. 2016 Jan 18;7:10305. doi: 10.1038/ncomms10305 (PMC4735631; doi:10.1038/ncomms10305)
Supplement: Supplementary Information — Supplementary Figures 1-9, Supplementary Table 1 and Supplementary References [file ncomms10305-s1.pdf]

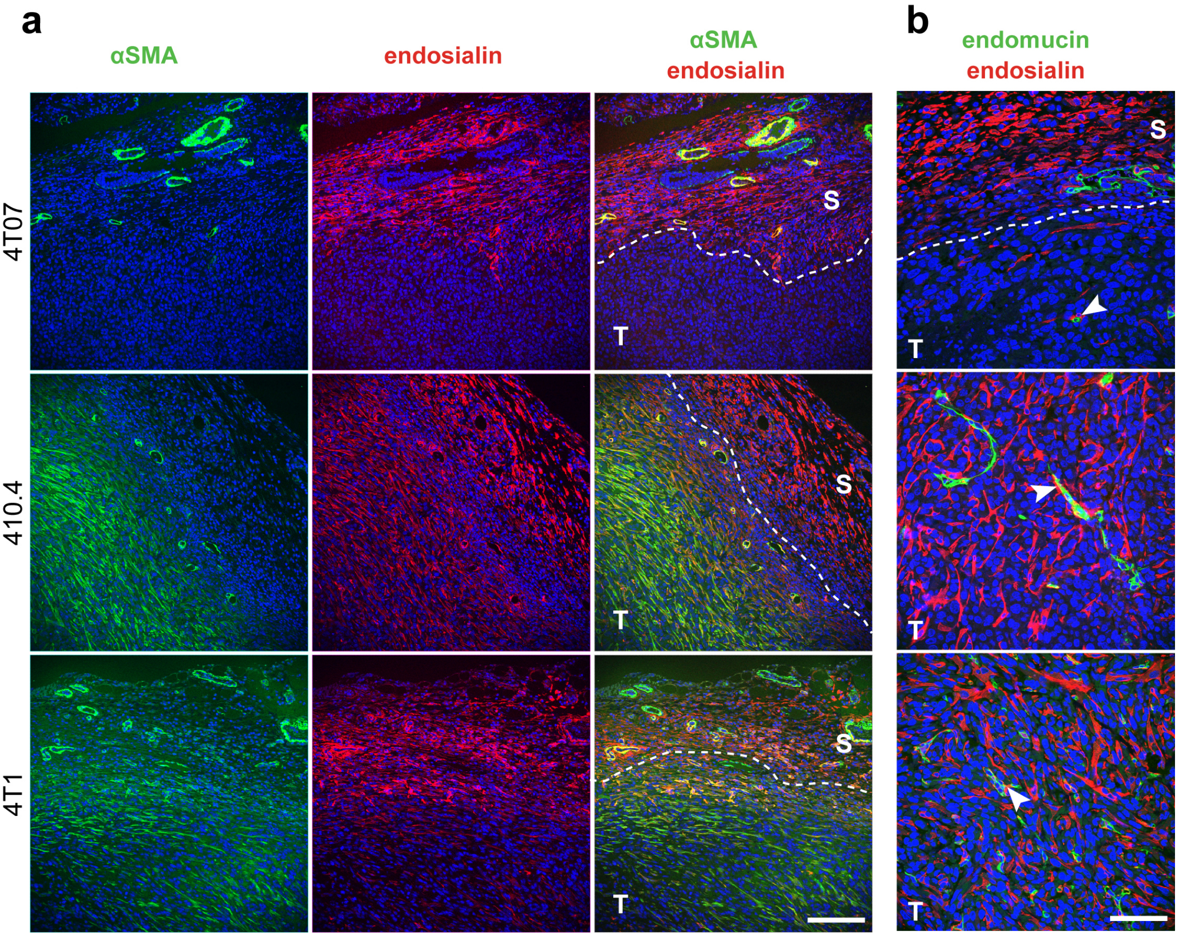

Supplementary Figure 1. Characterisation of the stroma in 4T1 series tumours. (**a**) Representative low power images of tumour sections described in Fig. 1a stained with αSMA-FITC (green) and endosialin followed by Alexa555-anti-rabbit-IgG (red). Nuclei were counterstained with DAPI (blue). Scale bar, 150 µm. Dashed line indicates border between tumour (T) and stroma (S). (**b**) As both endosialin and αSMA are also expressed by tumour pericytes[1], sections shown in panel (a) were additionally stained with the endothelial marker endomucin followed by Alexa633-anti-rat-IgG (green) and endosialin followed by Alexa555-anti-rabbit-IgG (red). Nuclei were counterstained with DAPI (blue). Representative confocal images are shown. Arrowheads indicate blood vessels. Scale bar, 150 µm. The low incidence of endosialin-positive cells associated with endomucin-positive vessels indicates that the infiltrating endosialin-positive cells are predominantly of fibroblast identity.

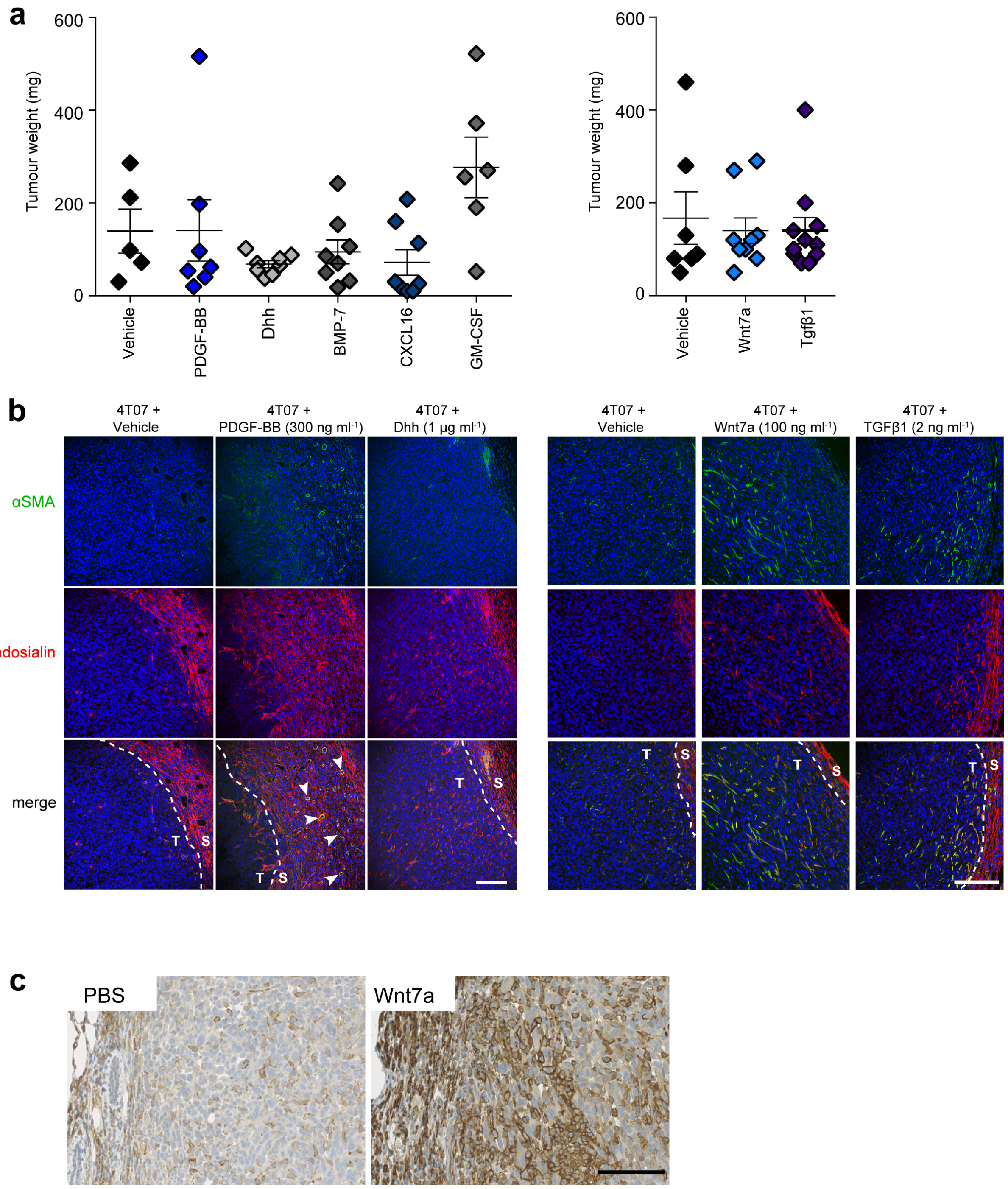

Supplementary Figure 2. Characterisation of 4T07 tumours supplemented with recombinant growth factors. 4T07 orthotopic tumours were treated *in vivo* with recombinant growth factors (see Fig. 2a,b). (**a**) Mean tumour weight at necroscopy ± SEM. Groups were compared with one-way ANOVA followed by Bonferroni post-test and no statistically significant differences were found. (**b**) Images shown in Fig. 2b separated to visualise individual antibody staining. Scale bar, 100 μm. (**c**) Sections from tumours injected with PBS or recombinant Wnt7a were stained for Wnt7a. Representative immunohistochemical images are shown. Scale bar, 100 μm.

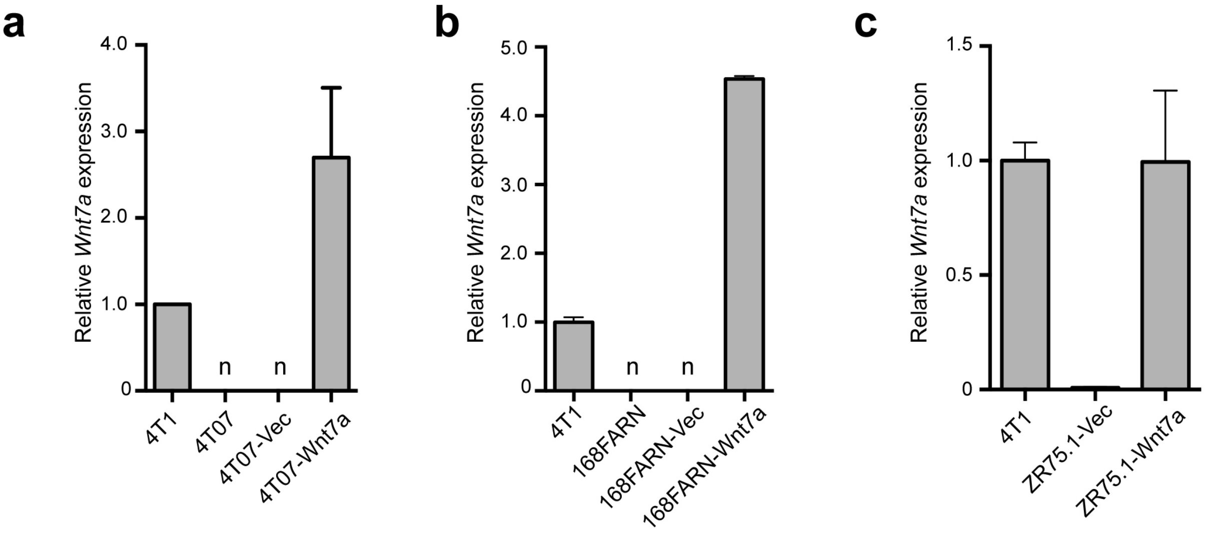

Supplementary Figure 3. Tumour cell lines with ectopic Wnt7a expression. *Wnt7a* mRNA expression assessed by qPCR. Data shown are mean RQ relative expression ± SEM. (**a**) 4T1, parental 4T07, 4T07-Vec and 4T07-Wnt7a cells. (**b**) 4T1, parental 168FARN, 168FARN-Vec and 168FARN-Wnt7a cells, and (**c**) 4T1, ZR75.1-Vec and ZR75.1-Wnt7a cells. n, non-detectable.

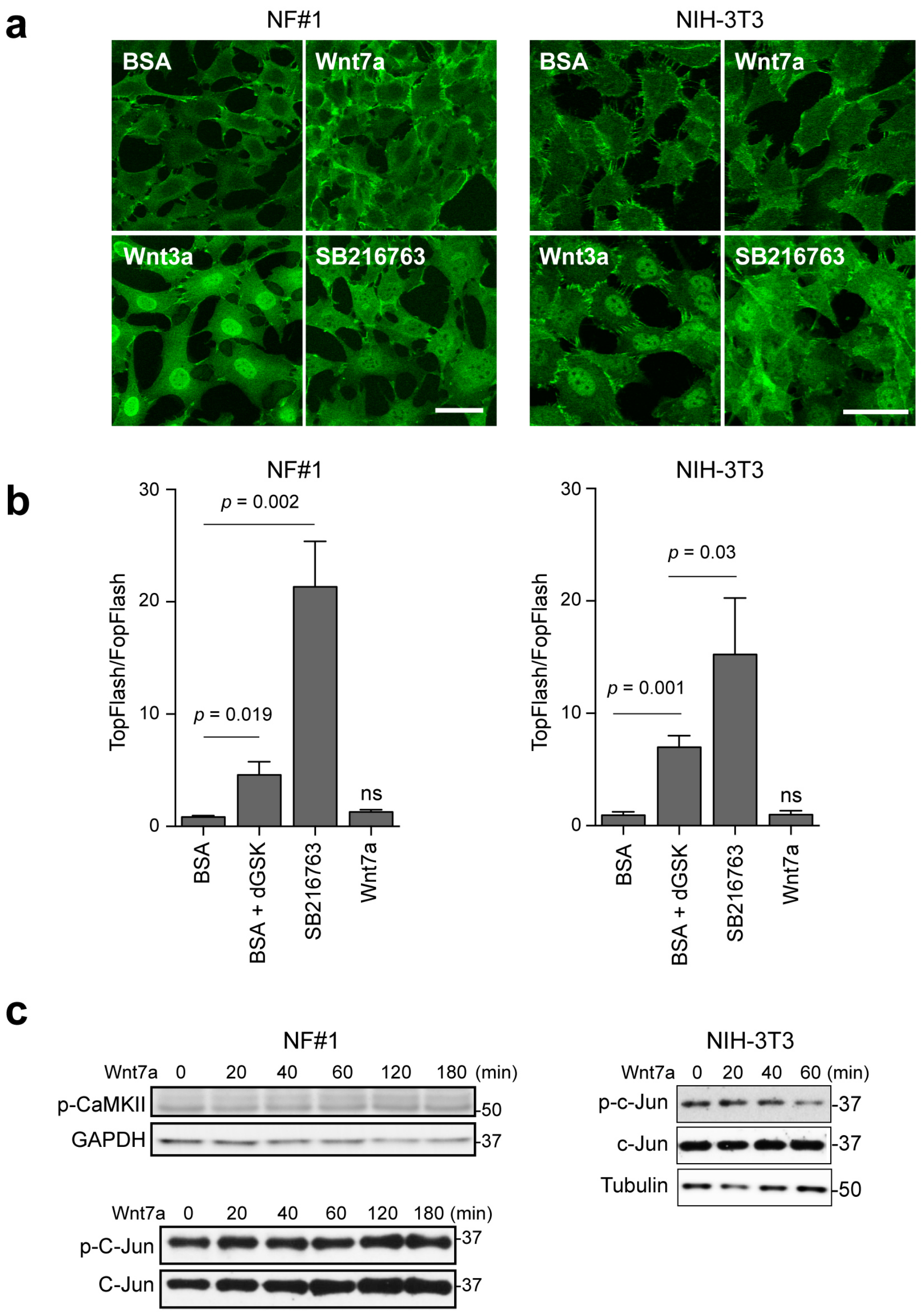

Supplementary Figure 4. Wnt7a signalling in NF#1 and NIH-3T3 fibroblasts in 2D culture. (**a**) NF#1 and NIH-3T3 fibroblasts were starved overnight in serum free Advanced DMEM and treated with BSA, Wnt7a (100 ng ml^{-1}), Wnt3a (50 ng ml^{-1}) or the GSK-3β inhibitor SB216763 (10 μM) for 5 hours. Cells were fixed and stained for β-catenin (green). Scale bar, 50 μm. (**b**) β-catenin transcriptional activation in NF#1 and NIH-3T3 fibroblasts was monitored using the TOPflash assay for 24 h. Comparisons to BSA were made using Student's *t*-test. (**c**) NF#1 and NIH-3T3 fibroblasts were starved overnight and then treated with Wnt7a (100 ng ml^{-1}) for the indicated times and subject to immunoblotting. Molecular size markers are in kDa.

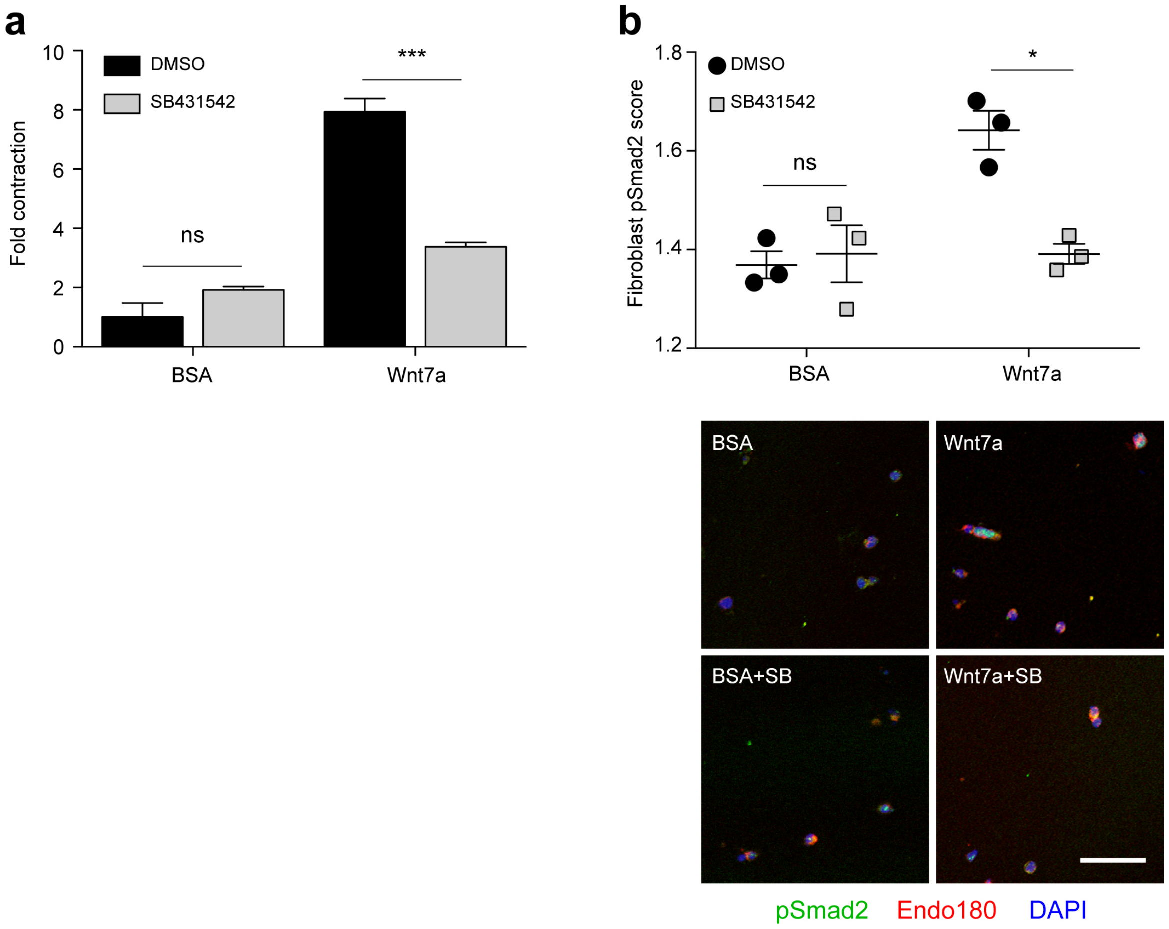

Supplementary Figure 5. Wnt7a increases NIH-3T3 fibroblast contractility in a TGFβ receptor-dependent manner. (**a**) NIH-3T3 fibroblasts embedded in a collagen/Matrigel gels were treated with BSA or Wnt7a (100 ng ml^{-1}) in presence or absence of ALK5 inhibitor SB43152 (10 μM) for 72 h. Data show quantification of matrix remodelling ± SEM. Groups were compared using two-way ANOVA followed by Bonferroni post-test. ***, $p < 0.001$. (**b**) Collagen gels from panel a were fixed, paraffin-embedded and sections were stained with antibodies against the pan-fibroblast marker Endo180 (*Mrc2*) followed by Alexa 555-anti-sheep-IgG (red) and phospho-Smad2 followed by Alexa 488-anti-rabbit-IgG (green). Nuclei were counterstained with DAPI. Upper panel, phospho-Smad2 staining in the fibroblast nuclei was scored as in Fig. 5b. Two-way ANOVA with Bonferroni post-test used to compare groups. *, $p < 0.05$. Lower panel, representative images are shown. Scale bar, 150 μm.

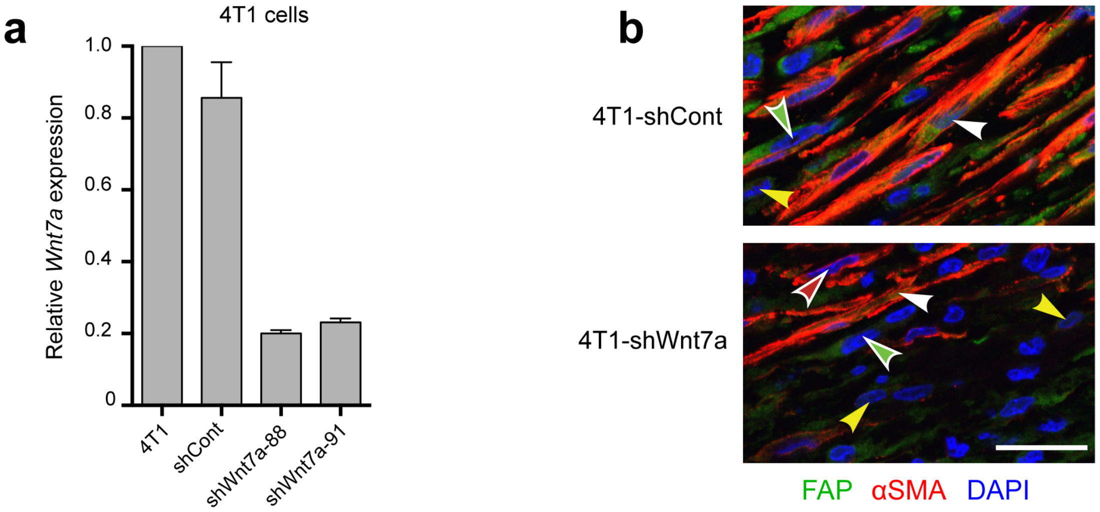

Supplementary Figure 6. Downregulation of Wnt7a expression in 4T1 cells. *Wnt7a* mRNA expression was assessed by qPCR in 4T1 parental cells, 4T1-shCont (SHC001V), 4T1-shWnt7a-88 (TRCN0000071788) and 4T1-shWnt7a-91 (TRCN0000071791) cells. Data shown are mean RQ relative expression ± SEM from 2 biological replicates. (**b**) FFPE sections from 4T1-shCont and 4T1-shWnt7a-88 tumours were stained for αSMA and fibroblast activation protein (FAP). Scale bar, 25 μm. Red arrowheads, αSMA+, FAP- fibroblasts; green arrowheads, αSMA-, FAP+ fibroblasts; white arrowheads, αSMA+, FAP+ fibroblasts; yellow arrowheads, αSMA-, FAP- fibroblasts.

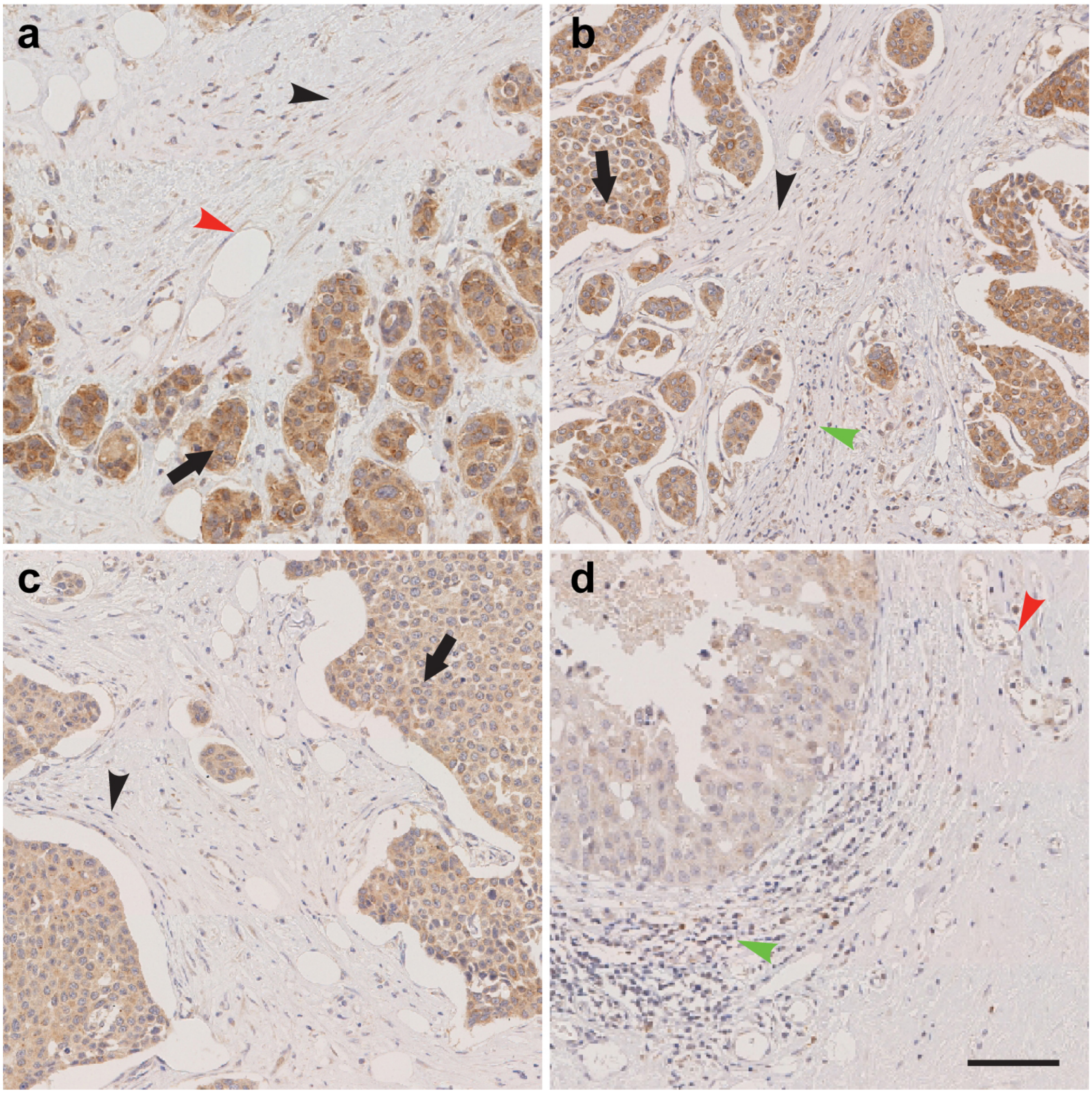

Supplementary Figure 7. Wnt7a protein localisation in human breast cancers. Shown are representative images from 4 different human breast cancers expressing high (**a**,**b**), moderate (**c**) and low (**d**) level Wnt7a as detected by immunohistochemical staining. Wnt7a protein is predominantly associated with tumour cell areas (arrows) with only low levels detected in the stroma. Fibroblast-rich (black arrowheads), vasculature (red arrowheads), and immune cell-rich (white arrowheads) stromal areas are indicated. Scale bar, 100 μm.

a

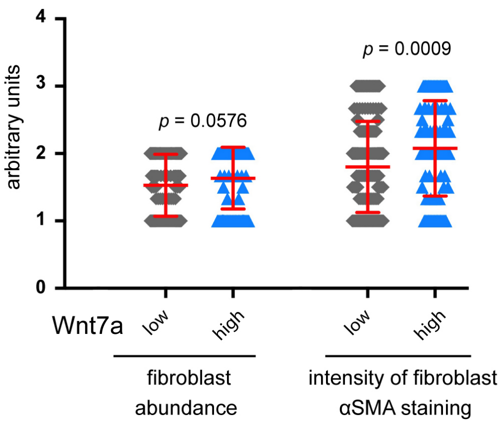

b

	Chang - fibroblast core serum response (F-CSR) signature , Spearman correlation<br>Module Score(s) = $\sum_{i=1}^{n} WiXi / \sum \lvert Wi \rvert$	
	***p* value**	**rho**
WNT7A	2.56 x 10^{-5}	0.244

c

	Calon - fibroblast TGFb response signature (F-TBRS), Pearson correlation	
	***p* value**	***r***
WNT7A	0.005	0.123

Supplementary Figure 8. High Wnt7a expression is associated with CAF activation and positively correlates with fibroblast serum response and TGFβ response signatures. (**a**) As described in Fig. 8, the 347 (Leeds) breast cancer TMA was stained for Wnt7a and αSMA. Wnt7a protein levels were scored as negative/low (n = 203) or high (n = 106). αSMA positive fibroblasts were scored for abundance as 1 (few fibroblasts) or 2 (abundant fibroblasts) and for aSMA expression as 1 (none/little), 2 (intermediate) or 3 (strong). Scores across the triplicate cores were averaged. Shown is the correlation between Wnt7a protein levels and abundance of αSMA-positive fibroblasts and between Wnt7a protein levels and intensity of αSMA staining (unpaired *t*-test). (**b**) Correlation of *WNT7A* expression with the fibroblast core serum response (F-CSR) signature described by Chang et al.[2] in the NKI295 dataset. Gene expression of the 442 probes from NKI295 dataset was used to calculate a module score for each sample. The module score is a weighted averaging as previously described[3], where n is the number of genes in the signature, *Xi* represents the normalised gene expression in the sample and gene-specific weights Wi are equal to +1 if the activated fibroblast centroid value of a gene >0, otherwise *Wi* are equal to −1. *WNT7A* gene expression was significantly associated with a high F-CSR signature (rho = 0.244, p = 2.56 x 10^{-5}; Spearman correlation). (**c**) Correlation of *WNT7A* expression with the fibroblast TGFβ response signature (F-TBRS) described by Calon et al.[4] in the TCGA dataset of 522 primary breast cancers. *WNT7A* gene expression was significantly associated with a high F-TBRS signature (r = 0.123, p = 0.005; Pearson correlation).

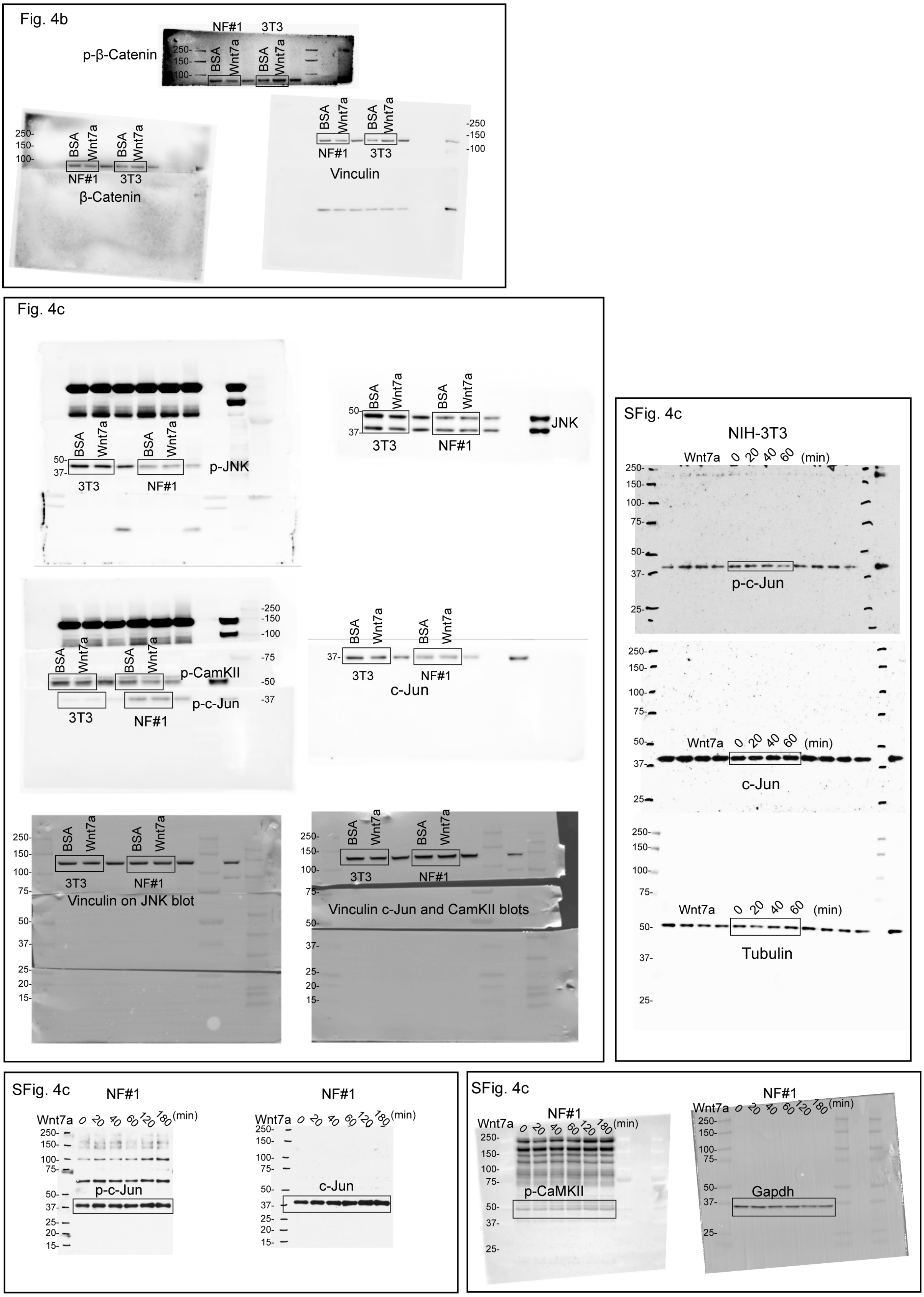

Supplementary Figure 9. Uncropped immunoblot images. Uncropped immunoblot images corresponding to Fig. 4b, Fig. 4c and Supplementary Fig. 4c. Molecular size markers are in kDa.

Supplementary Table 1 Secreted factors differentially upregulated in 4T1/401.4 tumour cells as compared to 4T07 tumour cells*

Gene symbol	Gene name	Fold change	Adjusted p-value
Spint1	serine protease inhibitor, Kunitz type 1	6.26	1.23E-07
Igfbp7	insulin-like growth factor binding protein 7	5.54	1.15E-05
Ace	angiotensin I converting enzyme 1, transcript variant 1	4.95	1.33E-04
Pdgfb	platelet-derived growth factor B chain	4.80	4.95E-04
Endod1	endonuclease domain containing 1	4.37	4.82E-05
Il23a	interleukin 23, alpha subunit p19	3.46	2.49E-03
Csf2	colony stimulating factor 2	3.01	3.34E-02
Stc1	stanniocalcin 1	2.94	3.75E-03
Pcolce2	procollagen C-endopeptidase enhancer 2	2.83	5.66E-04
Pdgfc	platelet-derived growth factor, C polypeptide	2.79	1.81E-04
Sfn	stratifin	2.79	2.36E-04
Inhba	inhibin beta-A	2.66	8.50E-04
Cd40	CD40 antigen, transcript variant 5	2.58	1.02E-02
Cxcl9	chemokine ligand 9	2.48	1.67E-02
Tgfbr3	transforming growth factor, beta receptor III	2.46	4.43E-05
Bmp1	bone morphogenetic protein 1	2.45	4.64E-04
Pdgfrl	platelet-derived growth factor receptor-like	2.43	7.03E-04
Nbl1	neuroblastoma, suppression of tumourigenicity 1	2.35	9.82E-03
Cxcl16	chemokine ligand 16	2.32	9.83E-03
Wnt7a	wingless-related MMTV integration site 7A	2.29	3.16E-03
Insl3	insulin-like 3	2.20	3.49E-03
Crlf1	cytokine receptor-like factor 1	2.15	2.21E-03
Fam132a	family with sequence similarity 132, member A	2.10	1.16E-03
Cxadr	coxsackie virus and adenovirus receptor, transcript variant 1	2.00	2.30E-03
Fgfbp1	fibroblast growth factor binding protein 1	1.90	1.32E-02
Dhh	desert hedgehog	1.89	5.98E-04
Rnase4	ribonuclease, RNase A family 4, transcript variant 1	1.86	3.62E-02
Ucn2	urocortin 2	1.84	6.36E-03
Pthlh	parathyroid hormone-like peptide	1.83	1.58E-02
Pros1	protein S	1.80	2.20E-02
5730469M10Rik	RIKEN cDNA 5730469M10 gene	1.71	2.00E-02

Supplementary Table 1 Secreted factors differentially upregulated in 4T1/401.4 tumour cells as compared to 4T07 tumour cells*

Gene symbol	Gene name	Fold change	Adjusted p-value
F13a1	coagulation factor XIII, A1 subunit	1.68	2.08E-02
Wisp1	WNT1 inducible signalling pathway protein 1	1.67	4.28E-02
Loxl4	lysyl oxidase-like 4	1.61	5.64E-03
Hhip	Hedgehog-interacting protein	1.59	4.57E-03
Cxcr3	chemokine receptor 3	1.58	4.50E-02
Adm	adrenomedullin	1.57	2.55E-02
Slit2	slit homolog 2	1.55	2.37E-03
Megf6	PREDICTED: multiple EGF-like-domains 6, transcript variant 4	1.53	7.03E-03
Rnpep	arginyl aminopeptidase (aminopeptidase B)	1.50	6.60E-04
Wnt7b	wingless-related MMTV integration site 7B	1.46	8.66E-03
Smpdl3b	sphingomyelin phosphodiesterase, acid-like 3B	1.46	3.10E-02
B4galt1	UDP-Gal:betaGlcNAc beta 1,4-galactosyltransferase, polypeptide 1	1.45	6.93E-03
Pdgfa	platelet-derived growth factor, alpha	1.45	7.76E-03
Pla2g7	phospholipase A2, group VII	1.45	1.61E-02
Acpl2	acid phosphatase-like 2	1.44	1.36E-02
Bmp7	bone morphogenetic protein 7	1.43	3.26E-02
Capg	capping protein, gelsolin-like, transcript variant 1	1.40	7.48E-03
Ccl25	chemokine ligand 25	1.36	5.25E-03
Fam20b	family with sequence similarity 20, member B	1.31	2.72E-02
Prss8	protease, serine, 8	1.29	3.34E-02
Psap	prosaposin	1.28	4.65E-02
Spp1	secreted phosphoprotein 1	1.25	4.40E-02
Hsd17b11	hydroxysteroid dehydrogenase 11	1.22	3.58E-02
Sdf2	stromal cell-derived factor 2	1.21	2.15E-02
2610507B11Rik	RIKEN cDNA 2610507B11 gene	1.21	8.98E-03

* Functional annotation using DAVID v6.7

Supplementary References

1. MacFadyen J, Savage K, Wienke D, Isacke CM. Endosialin is expressed on stromal fibroblasts and CNS pericytes in mouse embryos and is downregulated during development. *Gene Expr Patterns* **7**, 363-369 (2007).

2. Chang HY*, et al.* Robustness, scalability, and integration of a wound-response gene expression signature in predicting breast cancer survival. *Proceedings of the National Academy of Sciences of the United States of America* **102**, 3738-3743 (2005).

3. Loi S*, et al.* PIK3CA mutations associated with gene signature of low mTORC1 signaling and better outcomes in estrogen receptor-positive breast cancer. *Proceedings of the National Academy of Sciences of the United States of America* **107**, 10208-10213 (2010).

4. Calon A*, et al.* Dependency of colorectal cancer on a TGF-beta-driven program in stromal cells for metastasis initiation. *Cancer cell* **22**, 571-584 (2012).
